# Nonlinear transient analysis of water lubricated bearing in the turbulent regime

**DOI:** 10.1038/s41598-024-78242-4

**Published:** 2024-11-06

**Authors:** Ravindra Mallya, Raghuvir Pai, Satish Shenoy Baloor

**Affiliations:** 1 Department of Aeronautical and Automobile Engineering, Manipal Institute of Technology, Manipal Academy of Higher Education, Manipal, Karnataka 576104 India; 2 Department of Mechanical and Industrial Engineering, Manipal Institute of Technology, Manipal Academy of Higher Education, Manipal, Karnataka 576104 India

**Keywords:** Nonlinear analysis, Stability, Turbulence, Water lubricated, Journal bearing, Engineering, Mechanical engineering

## Abstract

Influence of turbulence on the stability characteristics of a water lubricated journal bearing is analyzed. Locus of the journal centre is plotted using the non – linear transient analysis method for different flow regimes of the bearing subjected to three different types of loading. The bearing groove angles of 18^o^ and 36^o^ is considered for the analysis. Turbulent coefficients are incorporated in the modified form of Reynolds equation which is solved using the 4th order Runge-Kutta method. The path traversed by journal centre is generated for different loading conditions and the stability of the bearing is examined. For periodic and fluctuating rotating loads, the journal centre traversed shorter path and reached stable position when the flow regime changed from laminar to turbulent.

## Introduction

**Figure 1:**
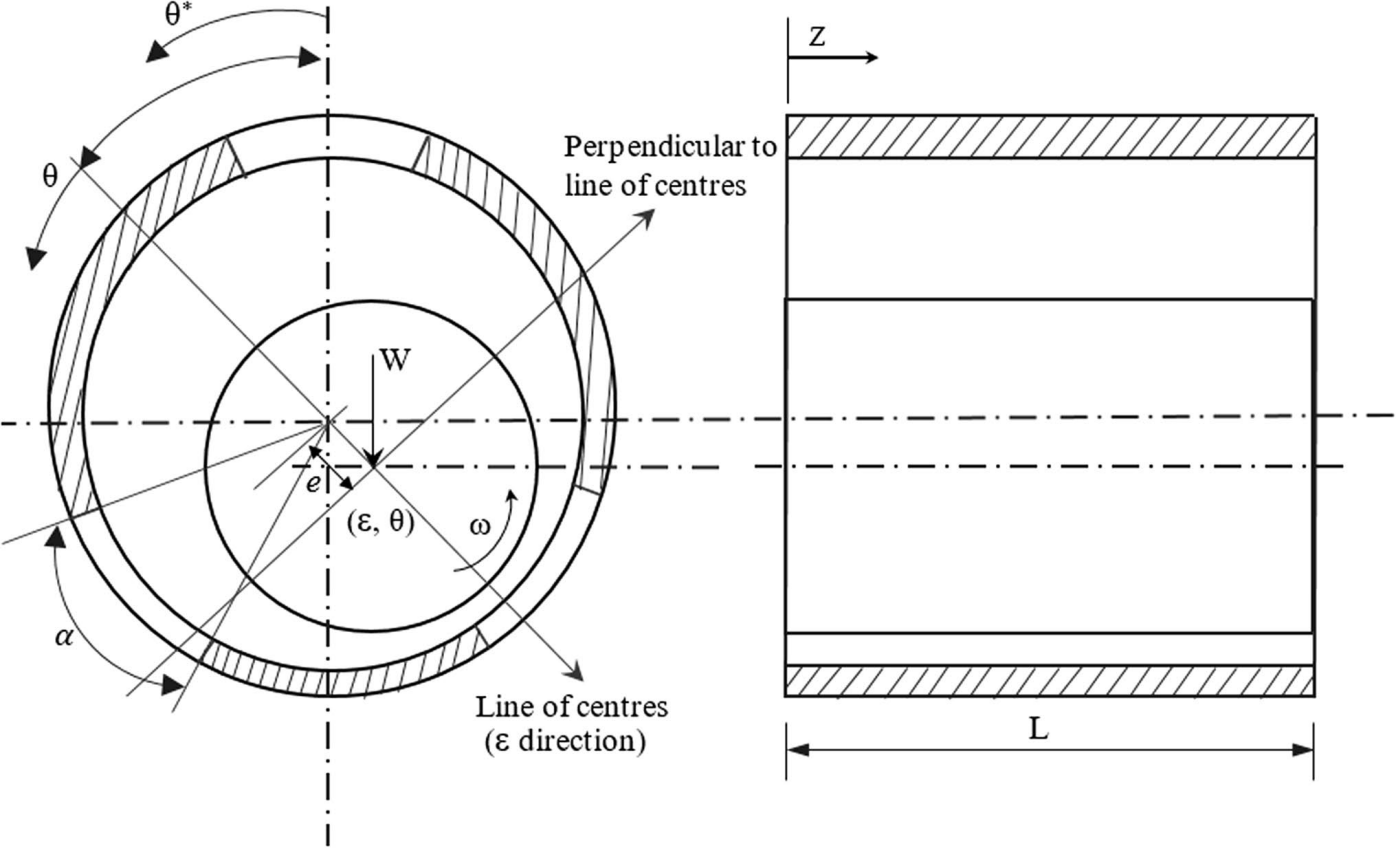
Schematic representation of 3 axial groove bearing and the coordinate system^12^.

The strict environmental protection laws, the increase in the ecological consciousness among the people, the requirement for an economic design for stern tube bearings have made water lubricated bearings popular as quoted by Litwin^1^. Cabrera et al^2^., states that bearings which use water as lubricant have alternate lands and grooves in the geometry, are commonly used in submarines, ships, small crafts and pumps. These type of bearings are generally manufactured with flexible rubber which is then bonded to a hard metal shell. The axial grooves of the bearing assist in the entry of the lubricant, water. The water enters in the axial direction and flows around the bearing to generate enough hydrodynamic pressure and exits through the other end. Fluid film journal bearings when operating under dynamic loading go through self-excited vibration known as whirl instability. Water which is an unconventional lubricant, has low dynamic viscosity and is used as lubricant in fluid film bearings in certain applications. The two common cause for turbulence as studied in the different literatures is the high speed of operation and low viscosity of the lubricant. The bearing stability is also one of the key factors for smooth operation. The amplitude of vibration may enhance and obstruct the safe working of bearing systems if the running conditions are not monitored. Turbulent flow or non – laminar flow is generated when the journal bearing system operate at high speeds or when the lubricant used are non – conventional fluids such as liquefied metal (sodium) or water as reported by Vinay Kumar^3^. The turbulent flow generated when low viscosity lubricants are used can cause instability in the bearings and hamper the smooth operation and lead to surface contact of shaft and the journal. A high journal speed and/ or low viscosity of fluid will increase the Reynolds number. Turbulent flow in the bearing occurs when the inertia forces of the fluid particles are much larger than the viscous forces. It is necessary to foresee the stability of the bearings for different regimes of flow and operational conditions. Researchers have investigated and analysed different bearing geometries and undertaken various theoretical and experimental studies on fluid film bearings to forecast the instability generated. Rao et al^4^., evaluated the dynamic coefficients and plotted the whirl orbits in their study on journal bearings using non – linear transient analysis for unbalance and impact excitations. The authors inferred that critical speeds predicted were same from linear and non – linear analysis. Lahmar et al^5^., used optimised short bearing theory on their non-linear dynamic analysis of an unbalanced rigid shaft, functioning both in the laminar and turbulent conditions. The author proposed a method which reduced the computation time for non-linear analysis without significant loss of accuracy. Ramesh et al^6^., found that surface roughness patterns of the bearings influence the stability of the elliptical bearings and claimed that transverse orientation of roughness showed improved stability than those with longitudinal roughness pattern. The study also concluded that a stable journal could become unstable when periodic loading was applied. Saha et al^7^., predicted the journal orbit for a double layered hydrostatic porous journal bearing with oil as the lubricant. The authors observed that the two layered bearings showed better stability for all speeds when compared to single layered bearings. The increase in the bearing feeding parameter reduced the stability of the bearing and bearings under highly loaded conditions were stable. Kushare et al^8^., found that, by increasing the wear depth parameter, there was increase in the stability threshold speed margin for both Newtonian and Non-Newtonian lubricants which were used in two lobed hybrid journal bearing. Lin et al^9^., considered the Stokes micro-continuum theory to analyse the short journal bearings with non-Newtonian couple stresses. The non-linear differential equations were solved using RK method and the journal centre plots illustrated that the influence of non-Newtonian fluid had better stability in comparison with Newtonian fluid. Wang et al^10^., investigated the dynamic performance of the rotor supported by axial groove long journal bearings. The influence of inlet pressure of the oil and instability threshold speed of the bearing system were analysed. It was observed that inlet pressure of lubricant had significant influence on the instability threshold speed and by repositioning the oil inlet location, improved the stability of the system. R S Pai et al^11,12^., theoretically analysed the stability characteristics of multiple axial grooved bearing system using non – linear transient analysis method. Two different boundary conditions, i.e., JFO and Reynolds were implemented in the analysis. Smaller groove angles favoured in making the bearing system more stable and for varying rotating load, the journal centre path was intricate, but did not demonstrate unstable behaviour. Majumdar et al^13^., investigated the stability of water lubricated bearings with multiple axial grooves. The authors found that bearing with smaller groove angle showed better stability characteristics, but increased in friction variable. Mongkolwongrojn et al^14^., investigated the importance of both bearing surface roughness and rheology of the lubricants on the stability of a rotor. Both perturbation and non –linear method was used in the analysis. Bearing having transverse roughness pattern and smaller L/D ratio exhibited better stability characteristics in the study. Laha et al^15^., studied the stability behaviour of porous hydrodynamic bearings using Wilson – *θ*method. The consequence of bearing feeding parameter, slenderness ratio on the stability of the rotor were investigated using non – linear transient analysis. Bearing with higher porosity resulted in poor stability performance and increase in slenderness ratio resulted in improved stability of the rotor. Raghunandana et al^16^., analysed the journal bearing system using non – Newtonian lubricants considering non – linear transient analysis. The non-Newtonian lubricant model developed by Dien and Elrod was incorporated in the analysis. It was observed that, the bearing showed improved stability when the lubricant with higher power law index was used and also for heavily loaded bearings. Vijayaraghavan et al^17^., analyzed the submerged journal bearing undergoing a unidirectional periodic load using non – linear analysis. The authors found that, for periodic loading, the journal attained a stable limit when the loading frequency was high. For smaller mass, the journal system attained a stable smaller limit cycle, and with smaller periodic load amplitude, the journal excursions were small. Ram et al^18^., in their investigation on rotor supported by hydrodynamic bearings with rough surfaces found that isotropic roughness reduced stability whereas transverse roughness enhanced the stability of the system. Bhudeeja & Verma^19^investigated the hybrid journal bearing lubricated with micropolar fluids for stability using non – linear transient method. Journal center paths have been plotted using the equations of motions to assess the stability of the bearing. The authors state that, even during high loading conditions on the bearing, the stability of the bearing can be enhanced by altering the lubricant flow rate and conclude that micropolar fluids increase the stability of the bearing at high loading conditions. Some & Guha^20^traced the journal center locus to determine the stability of 2 layered porous journal bearing in their analysis. Comparison between linear and non – linear stability analysis has also been undertaken considering the critical mass parameter and whirl ratio. The authors found that, bearing stability enhanced when the eccentricity ratio and the L/D ratio was high. The authors conclude that Non-linear method is more accurate and gives better results. Mallisety et al^21^. studied the stability of gas lubricated, double layered porous bushing. The journal centre trajectories were plotted to ascertain the stability of the bearing system in which velocity slip in the interface between the film and porous region was considered. The Authors established that, with increase in the eccentricity ratio, the stability of the bearing enhanced and the slip parameters such permeability factor and slip coefficient reduced the stability. Xiang et al^22^. investigated the nonlinear dynamic performance of water lubricated bearings considering the influence of friction force and thermal effect. The researchers inferred that the friction coefficient influenced the dynamic behavior of the lubricated rotor. The authors do not recommend the use of perturbed method of analysis of these bearings when the unbalance ratio is very high. Miyanaga et al^23^. analyzed the journal bearing using mass conservative cavitation model using both linear and non – linear transient study and developed stability maps. The journal centre loci plotted for different shaft speed and eccentricity ratios predicted the stability of the bearing. It was observed that cavitation pressure influenced the stability of the bearing significantly. The construction features and the working principle of water lubricated bearings is different from the conventional journal bearings. Schematic representation of 3 axial groove bearing and the coordinate system is shown in Fig. 1. The non-linear stability studies of water lubricated bearings is limited and has not been explored adequately by the researchers. In the present work, the journal locus orbits of axial groove water lubricated bearings are plotted for different degrees of turbulent conditions. Three different type of loading situations i.e., constant load condition, unidirectional periodic load and rotating load varying with magnitude are considered with two different groove angles of the bearing.

## Theoretical analysis

The numerical method considered in Majumdar and Brewe^24^ is implemented in establishing the stability of the bearing for different degrees of turbulent conditions.

The general Reynolds equation for pressure distribution in the bearing clearance is given as follows

1 $$\frac\partial{\partial x}\left(h^3\frac{\partial p}{\partial_x}\right)+h^3\frac{\partial^2p}{\partial z^2}=6\eta U\frac{\partial h}{\partial x}+12\eta\frac{\partial h}{\partial t}$$

The linearized turbulent model developed by Ng and Pan^25^ is used in the present analysis.

To simulate the influence of turbulence, the general form of Reynolds equation is modified by introducing turbulent coefficients as suggested in Taylor and Dowson^26^.

The coefficients *k*_θ_ and *k*_*z*_as suggested in^26^ is given as

$$k_\theta=12+k_x\left(Re_L\right)^{n_x}$$

2 $$k_z=12+k_{zz}\left(Re_L\right)^{n_z}$$

The altered Reynolds Eq. 1 in the general form becomes,

3 $$\frac\partial{\partial x}\left[\frac1{k_\theta}h^3\frac{\partial p^\ast}{\partial x}\right]+\frac\partial{\partial z}\left[\frac1{k_z}h^3\frac{\partial p^\ast}{\partial\theta}\right]=\frac12U\eta\frac{\partial h}{\partial x}+\eta\frac{\partial h}{\partial t}$$

After the following substitutions,

$$\theta=\frac xR,\;\;\;\overline z=\frac zL,\;\;\overline p=\frac p{p_s},\;\;\tau=\omega_pt,\;\;\overline h=\frac hC=1+\varepsilon\;cos\mathit{\left({\theta^\ast-\psi}\right)}$$

Non – dimensional form of the Reynolds equation with turbulent coefficients is shown in Eq. 4.

4 $$\frac{12}{{k_\theta }}\left[ {{{\bar h}^3}\frac{{{\partial^2}\bar p}}{{\partial {\theta^2}}} + 3{{\bar h}^2}\frac{\partial \bar h}{{\partial \theta }}\frac{\partial \bar p}{{\partial \theta }}} \right] + \frac{12}{{k_z}}{\left( \frac{R}{L} \right)^2}{\bar h^3}\frac{{{\partial^2}\bar p}}{{\partial {{\bar z}^2}}} - \Lambda \frac{\partial \bar h}{{\partial \theta }} - 2\lambda \Lambda \frac{\partial \bar h}{{\partial \tau }}$$

Equation 4 can be rewritten as

5 $$\begin{array}{l} \frac{12}{{k_\theta }}\left[ {{{\bar h}^3}\frac{{{\partial^2}\bar p}}{{\partial {\theta^2}}} + 3{{\bar h}^2}\left( { - \varepsilon \sin \left( {{\theta^* } - \psi } \right)} \right)\frac{\partial \bar p}{{\partial \theta }}} \right] + \frac{3}{{k_z}}{\left( \frac{D}{L} \right)^2}{{\bar h}^3}\frac{{{\partial^2}\bar p}}{{\partial {{\bar z}^2}}} \hfill \\ - \Lambda \left( { - \varepsilon \sin \left( {{\theta^* } - \psi } \right)} \right) - 2\lambda \Lambda \left( {\dot \varepsilon \cos \left( {{\theta^* } - \psi } \right)} \right) = 0 \hfill \\ \end{array}$$

Where $$\Lambda = \frac{6\eta \omega }{{{{\left( \frac{C}{R} \right)}^2}{p_s}}}$$ and $$\dot \varepsilon = \partial \varepsilon /\partial \tau$$

Finite difference method is used in solving Eq. 5. The parameters $\dot \varepsilon$ and $\dot \phi$ are initialized as zero and the hydrodynamic forces $\bar F_r$ and $\bar F_\theta$ are calculated.

6 $${\bar F_r} = \left( {\frac{{F_r}}{{LD{p_s}}}} \right) = - \int\limits_0^1 {\int\limits_0^{2\pi } {{\bar p}} } \cos \left( {{\theta^* } - \psi } \right)d\theta \,d\bar z$$

$${\bar F_\theta } = \left( {\frac{{F_\theta }}{{LD{p_s}}}} \right) = - \int\limits_0^1 {\int\limits_0^{2\pi } {\bar p} } \sin \,\left( {{\theta^* } - \psi } \right)d\theta \,d\bar z$$

The Eqs. 7 and 8 are used to compute the values of $\varepsilon ,\,\,\phi ,\,\,\dot \varepsilon$ and $\dot \phi$ for the succeeding time interval.

7 $$MC\left[ {\frac{{{d^2}\varepsilon }}{{d{t^2}}}\,\, - \,\,\varepsilon {{\left( {\frac{{d\varphi }}{{dt}}} \right)}^2}} \right]\,\,=\,\,{F_r}\,\,+\,\,W\cos \varphi$$

8 $$MC\left[ {\varepsilon \frac{{{d^2}\varphi }}{{d{t^2}}}\,\,+\,\,2\left( {\frac{{d\varphi }}{{dt}}} \right)\left( {\frac{{d\varepsilon }}{{dt}}} \right)} \right]\,\,=\,\,{F_\theta } - W\sin \varphi$$

Equations 7 and 8 in the dimensionless form are as follows

9 $$\overline M\ddot\varepsilon-\overline M\varepsilon\dot\phi^2-\frac{{\overline F}_r}{{\overline W}_o}-\cos\phi=0$$

10 $$\overline M\varepsilon\ddot\phi+2\overline M\dot\varepsilon\dot\phi-\frac{{\overline F}_\theta}{{\overline W}_o}+\sin\phi=0$$

Where $$\bar M = \frac{{MC{\omega^2}}}{{LD{p_s}}}$$ and $${\overline W}_o=\frac{W_o}{LDp_s}$$

The non – dimensionalized differential Eqs. 9 and 10 in terms of $\varepsilon$ and $\phi$ are solved considering the 4th order Runge – Kutta method, to calculate the variables $\Omega,\,\,\overline M,\,\,{\overline F}_r$ and${\overline F}_\theta$ . The polar graph of $\varepsilon$ and $\phi$with respect to time traces the path of the journal midpoint. The plot generated is used to analyse the stability characteristics of the moving journal. Three different conditions of load as stated in^24^ is implemented in the study. Table 1shows the values of turbulent coefficients^26^ used in the present analysis.

**Table 1:** Turbulent coefficients values from reference^26^.

	*k*_*x*_	*n*_*x*_	*k*_*zz*_	*n*_*z*_
*Re*_*L*_≥ 50,000	0.0388	0.80	0.0213	0.80
10,000 ≥ *Re*_*L*_< 50,000	0.0250	0.84	0.0136	0.84
5,000 ≥*Re*_*L*_< 10,000	0.0250	0.84	0.0088	0.88
*Re*_*L*_< 5,000	0.0039	1.06	0.0021	1.06

### Unidirectional constant load

For specific values of eccentricity, $\varepsilon = 0.8$, Mass parameter = 5, and whirl ratio, $\Omega = 0.5$, the steady state hydrodynamic forces are computed as stated in Majumdar^24^. The variables, eccentricity and the attitude angle are computed for a predefined time interval using the equations of motion 7 and 8. The latest values of eccentricity ratios and attitude angles are substituted in Eq. 5 to calculate the next set of hydrodynamic forces. The hydrodynamic forces, steady state force, mass parameter value and whirl ratio are computed from Eqs. 9 and 10. The trajectory of journal centre is then plotted to illustrate the motion of the shaft.

### Periodic load (unidirectional)

The load acting on the bearing is considered as the sinusoidal function of steady state load as given in the Eq. 11.

11 $$\overline W={\overline W}_o\left[1+\sin\left(\frac T2\right)\right]$$

The load applied on the bearing is calculated for every time step. Initially the steady state load ${\overline W}_o$ is computed for $\varepsilon = 0.8$ , Mass parameter = 5 and $\Omega = 0.5$as stated in Majumdar^24^. Implementing the hydrodynamic forces ${\overline F}_r$, ${\overline F}_\theta$ the load $\overline W$ , in Eqs. 9 and 10, parameters like eccentricity ratio, attitude angle and their respective derivatives with respect to time are computed. The method is repeated to plot the different values of eccentricity ratio, attitude angle and their derivatives for predefined time step and the stability of the system can be evaluated.

### Fluctuating rotating load

The load data stated in^11^ is incorporated in the analysis. Table 2 lists the load data for every 10^o^ crank angle.

**Table 2:** Non – dimensional fluctuating rotating load from reference^11^.

Sl. No.	Crank rotation angle (^o^)	Cam lift (mm)	Fluctuating load values (*N*)	Non – dimensionalized load values
1	0	8	3980	18.84057
2	10	7.935	3947.66	18.68749
3	20	7.741	3851.15	18.23060
4	30	7.427	3694.93	17.49111
5	40	7.003	3483.99	16.49256
6	50	6.484	3225.79	15.27028
7	60	5.889	2929.78	13.86901
8	70	5.237	2605.41	12.33351
9	80	4.551	2264.13	10.71793
10	90	3.852	1916.37	9.07173
11	100	3.162	1573.09	7.44673
12	110	2.501	1244.25	5.89003
13	120	1.889	939.78	4.44872
14	130	1.342	667.65	3.16051
15	140	0.875	435.32	2.06068
16	150	0.499	248.23	1.17518
17	160	0.224	111.44	0.52753
18	170	0.056	27.86	0.13188
19	180	0.001	0.49	0.0023

The load data obtained is from the analysis conducted on a radial piston pump with a discharge rate of 1 × 10 ^−3^ m^3^/min at 30 MPa pressure. The vertical shaft speed was 1450 rpm. The maximum load on the piston is 3980 N, and the peak displacement of the cam is 8 mm. The lift of the cam for every 10^o^ rotation of the cam was graphically determined. The load on the bearing is assumed to be least when the crank angle is at 180^o^. The non – dimensionalized load values were repeated in a reverse order for the subsequent stroke. A time step of $\Delta T = {\pi \mathord{\left/ {\vphantom {\pi {18}}} \right. \kern-0pt} {18}}$ is considered.

## Solution procedure

**Figure 2:**
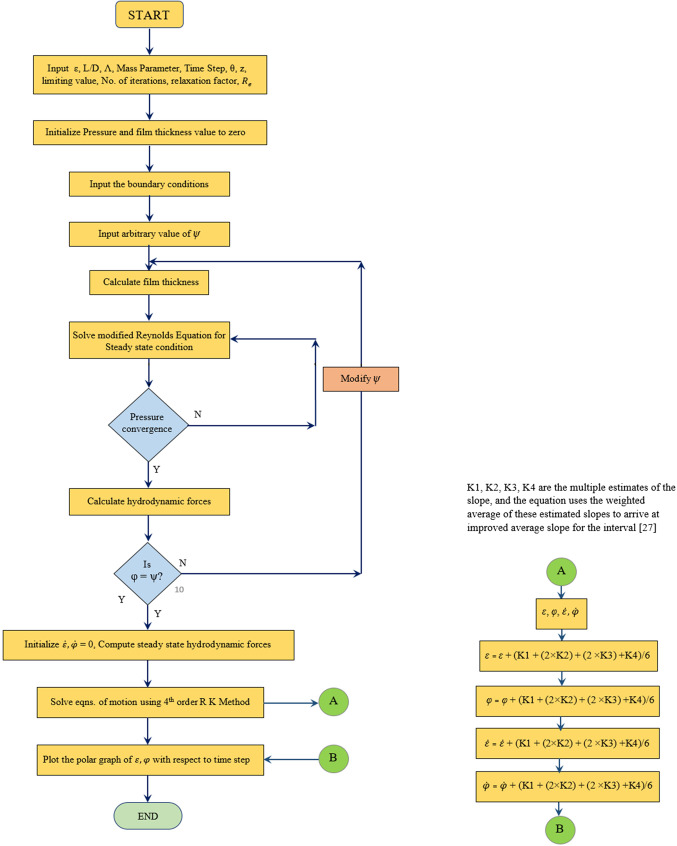
Flow chart of the solution procedure.

A MATLAB program is established to estimate the non – dimensional dynamic pressure. The Reynolds equation which has been incorporated with the turbulent coefficients is used for computing the hydrodynamic pressure. The convergence of the arbitrary attitude angle and calculated attitude angle is achieved initially. The steady state pressure convergence is then achieved for a predefined accuracy. The hydrodynamic forces are calculated using a fixed value of eccentricity ratio ($\varepsilon = 0.8$), mass parameter ($\overline M=5$), whirl ratio ($\Omega = 0.5$) and for a Reynolds number considering the turbulent regime. Utilizing the computed force values, and implementing it in the fourth order R.K. method^27^, the values of eccentricity ratio and attitude angles for each time step is found through Eqs. 9 and 10. The procedure is repeated for a given iteration value. The calculated values of eccentricity ratio $(\varepsilon )$ and attitude angles $(\phi )$ are plotted for each time interval, so as to assess the stability of the journal bearing. A flow chart of the solution procedure is shown in the Fig. 2

## Boundary conditions

**Figure 3:**
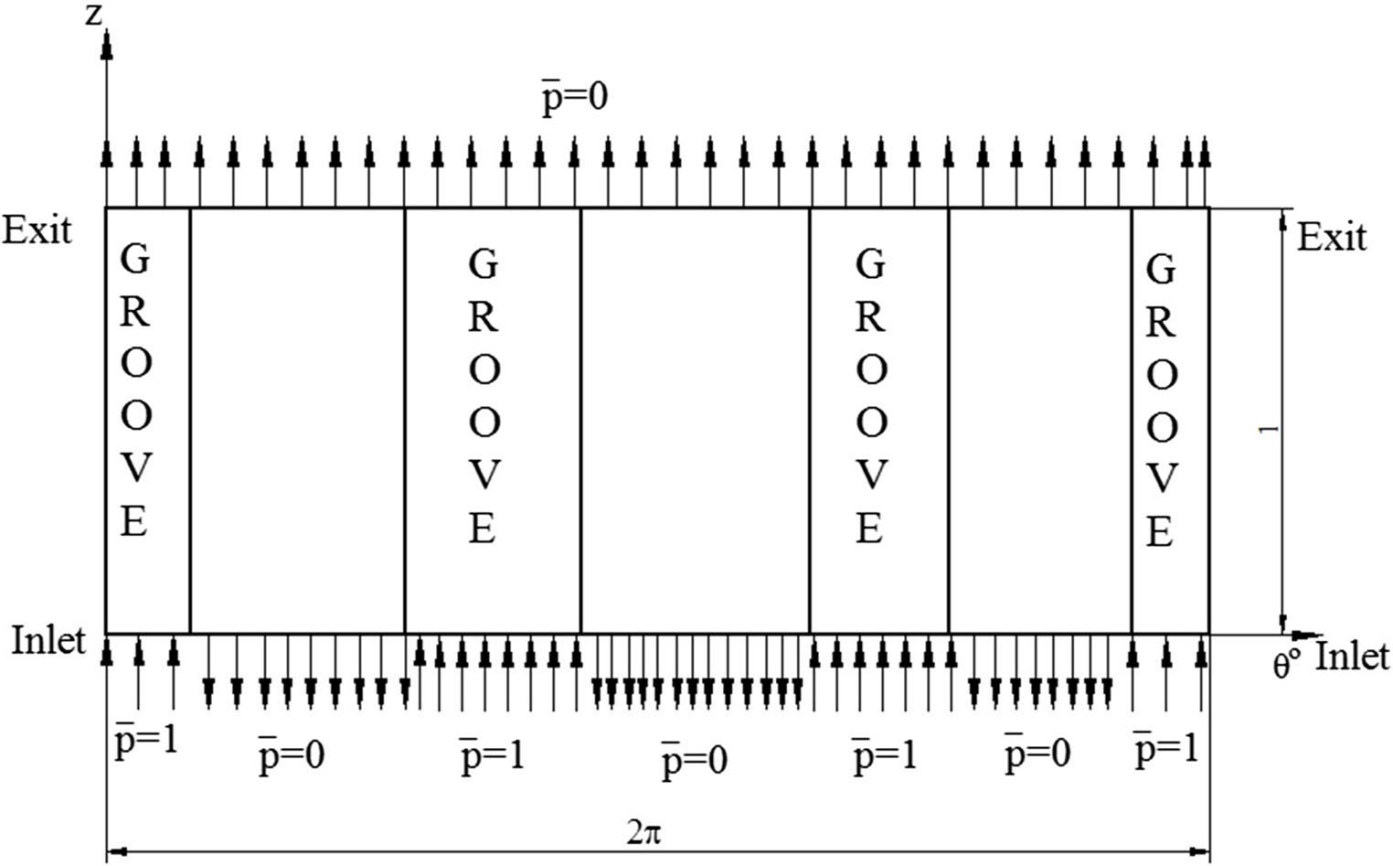
Boundary conditions shown for the axial groove bearing (unwrapped view)^13^.

In the current work, the boundary conditions stated in^13^ for a bearing with three axial grooves is implemented. The boundary conditions incorporated is illustrated in the Fig. 3. At the inlet edge, where the lubricant enters the bearing, $\bar z = 0$, the non – dimensional pressure at the groove is considered constant and taken as 1, i.e. $\bar p = 1$. At the land region, or the non – groove areas, the pressure is considered as zero, $\bar p = 0$. At the outlet side of the bearing, where $\overline z=1$, the non – dimensional pressure is considered as zero, $\bar p = 0$ for both the land and the groove regions. Cavitation is allowed to take place in the lubricant film at ambient pressure by considering the computed negative pressure equal to zero. Reynolds boundary condition $\bar p = \frac{\partial \bar p}{{\partial \theta }} = 0$ is implemented in the analysis.

## Validation

**Figure 4:**
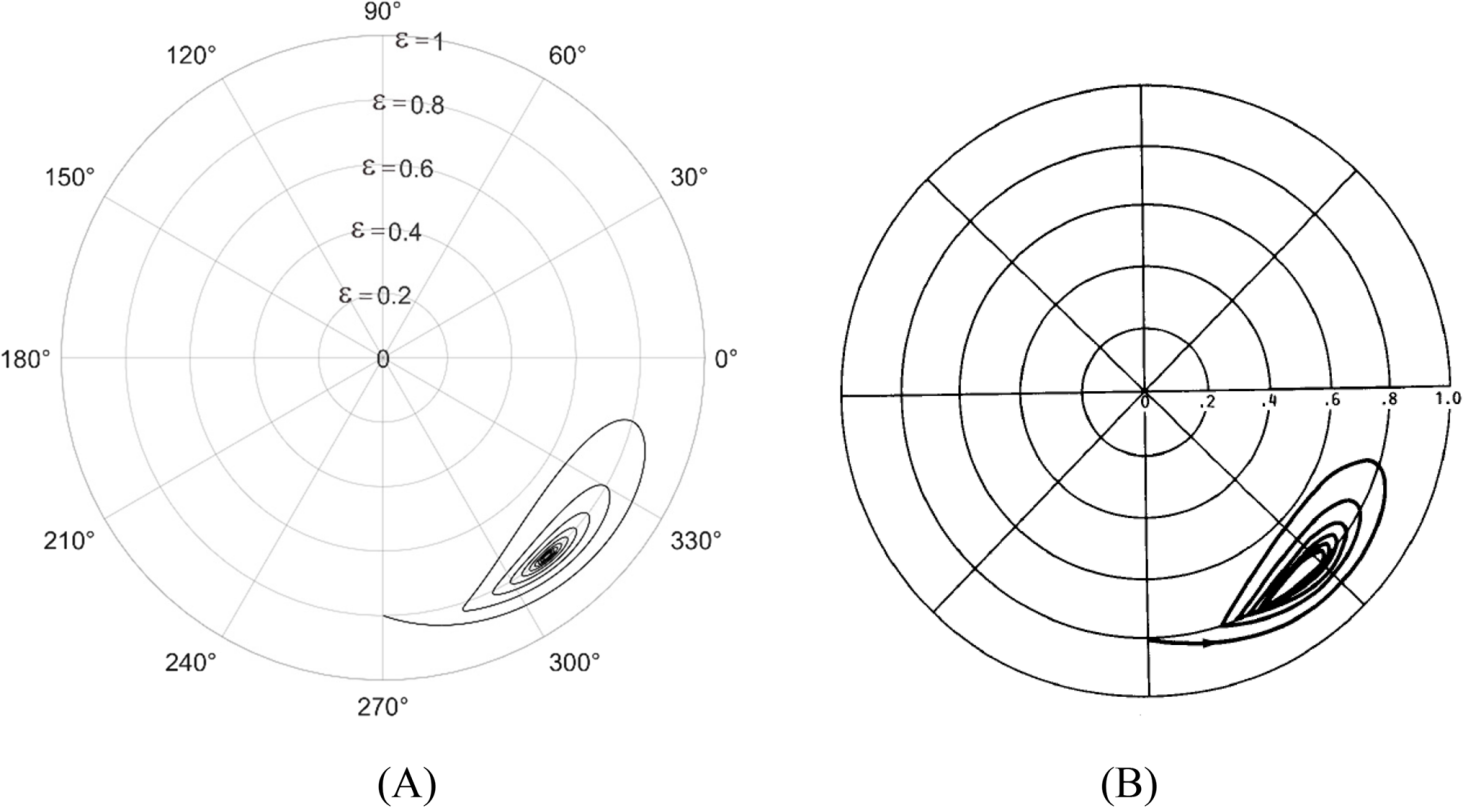
Comparison of the trajectory of the shaft centre for constant loading (unidirectional). $\:\frac LD=1,\:\epsilon=0.8,\:\overline M=5,\;\Omega=0.5$ (**A**) Present analysis (Re = 1), (**B**) Reference^24^ .

**Figure 5:**
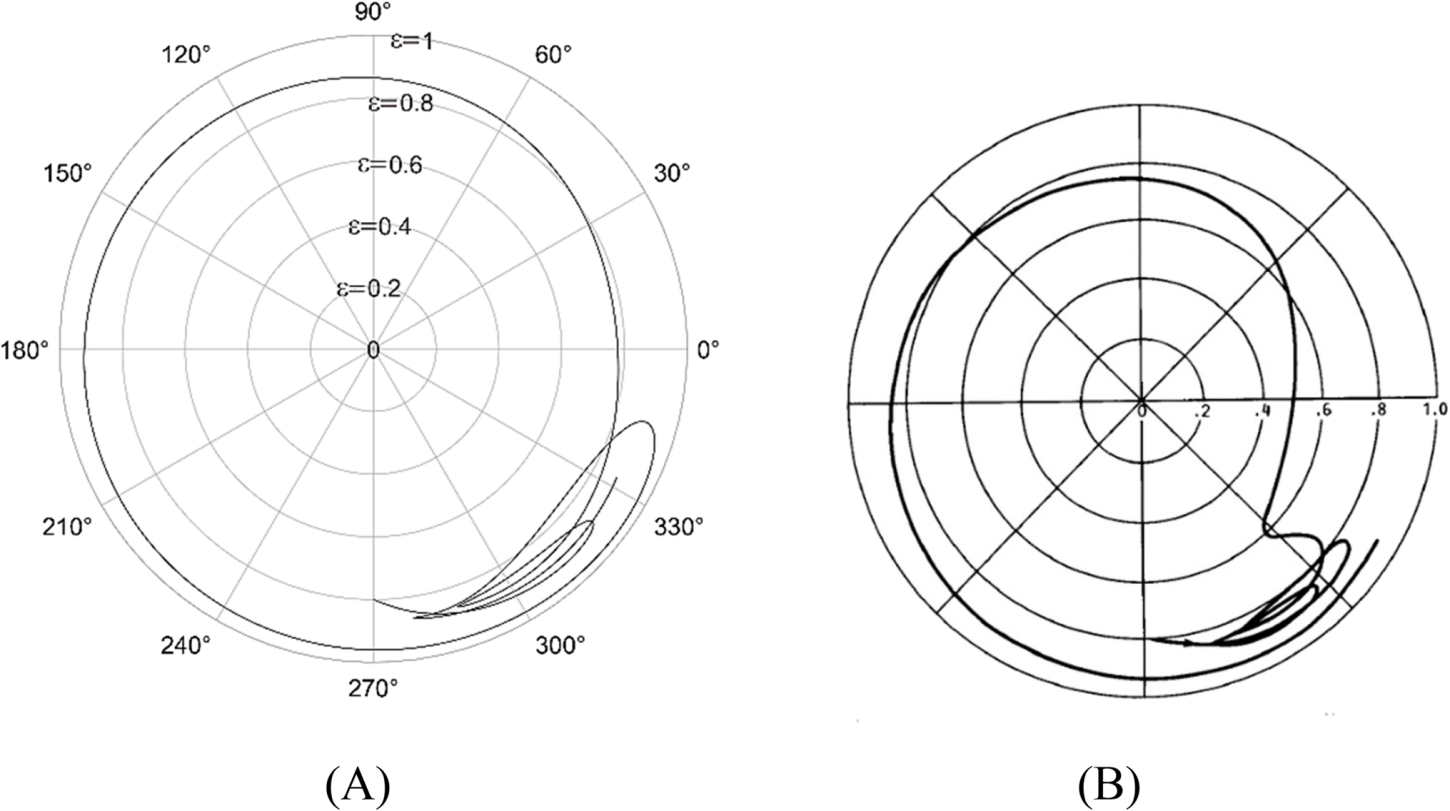
Comparison of the trajectory of the shaft centre for periodic load (unidirectional). $\:\frac LD=1,\:\epsilon=0.8,\:\overline M=5,\:\Omega\:=0.5$ (**A**) Present analysis (Re = 1), (**B**) Reference^24^.

The solution to the modified Reynolds equation, is accomplished using the finite difference method. To validate the solution, plots from the Majumdar and Brewe^24^is compared with the present analysis. The lubricant flow regime is considered to be laminar and therefore Reynolds number, Re = 1 is employed. The generated plots is compared with^24^ and is found to be in good agreement as seen from Figs. 4 and 5.

## Results and discussions

The journal locus for groove angle 36^o^ and 18^o^ are plotted in the Figures from Figs. 6, 7, 8, 9, 10, 11, 12, 13, 14, 15, 16, 17, 18, 19, 20, 21, 22 and 23. The Reynolds number considered are 4000, 16,000 and 55,000 which are in the different turbulent regimes. The three types of loading considered are constant load, sinusoidal function of steady state load and rotating loads with varying magnitude.

**Figure 6:**
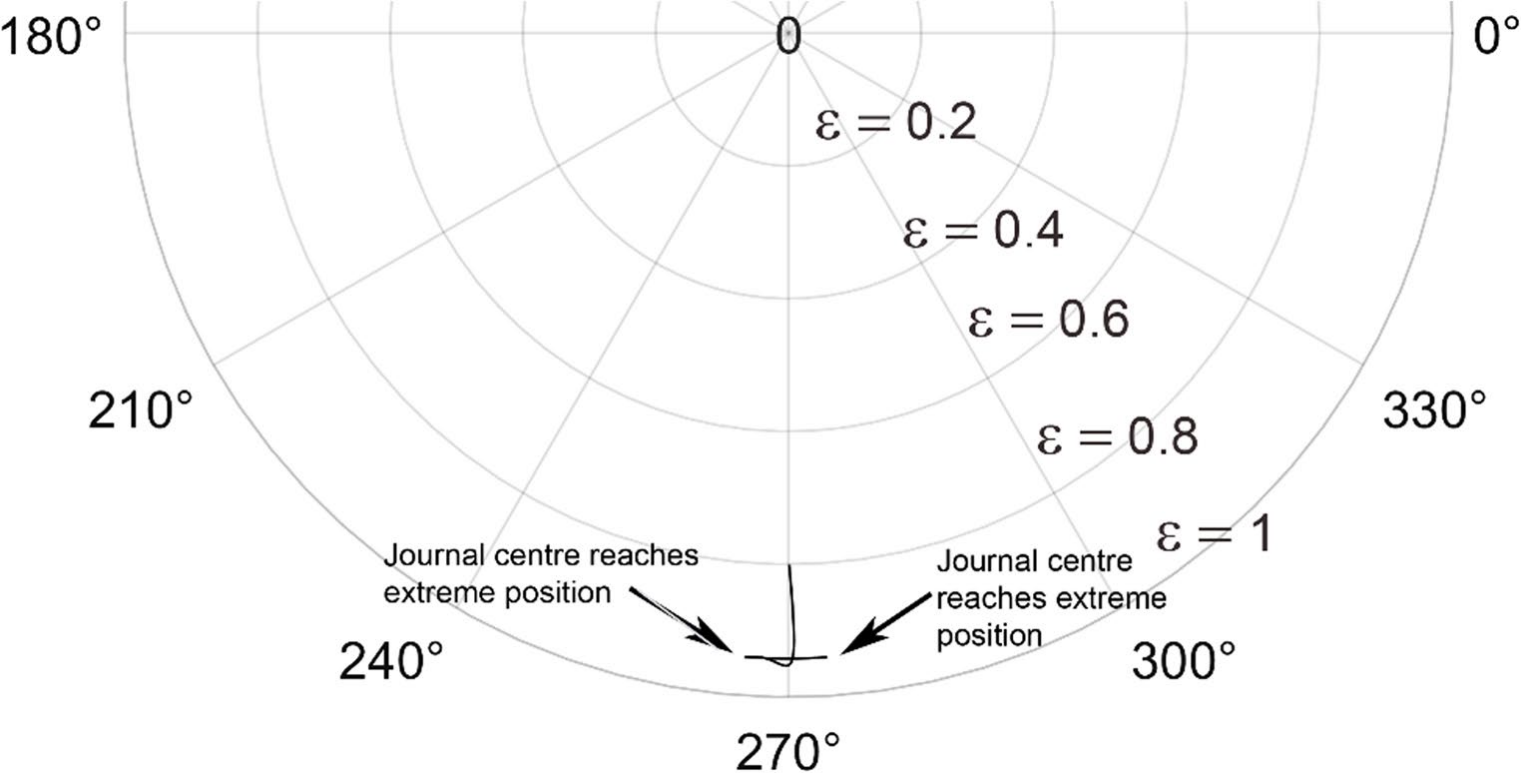
Journal centre plot for constant load (unidirectional), Re = 4000, $\Lambda=1,\,\varepsilon=0.8,\,\,\,\overline M=5,\,\Omega=0.5,\;\mathrm{groove}\;\mathrm{angle}=18^\circ$.

**Figure 7:**
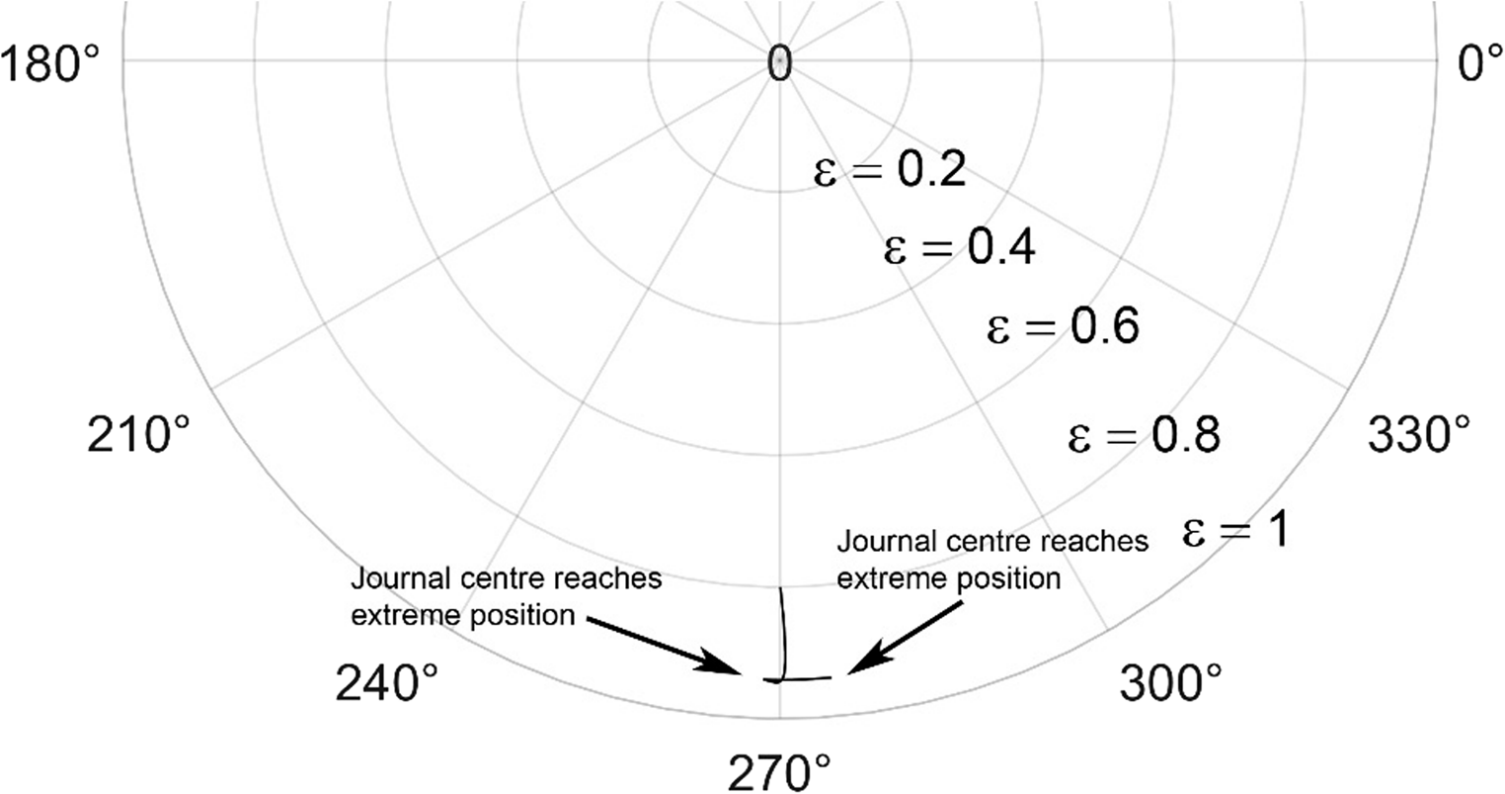
Journal centre plot for constant load (unidirectional), Re = 16,000, $\Lambda=1,\,\varepsilon=0.8\,\,\overline M=5,\,\Omega=0.5,\;\mathrm{groove}\;\mathrm{angle}=18^\circ$.

**Figure 8:**
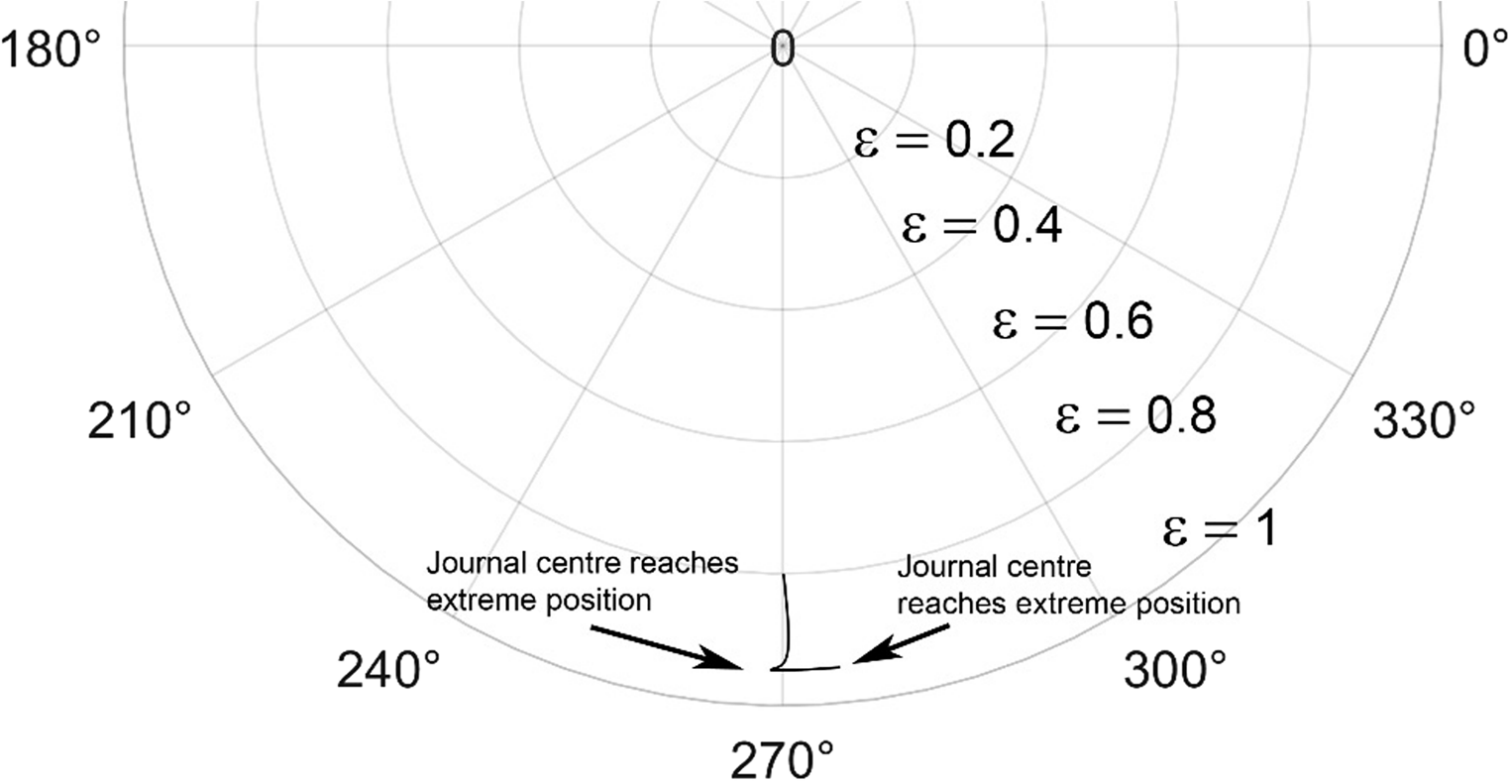
Journal centre plot for constant load (unidirectional), Re = 55,000, $\Lambda=1,\,\varepsilon=0.8,\overline M=5,\,\Omega=0.5,\;\mathrm{groove}\;\mathrm{angle}=18^\circ$.

**Figure 9:**
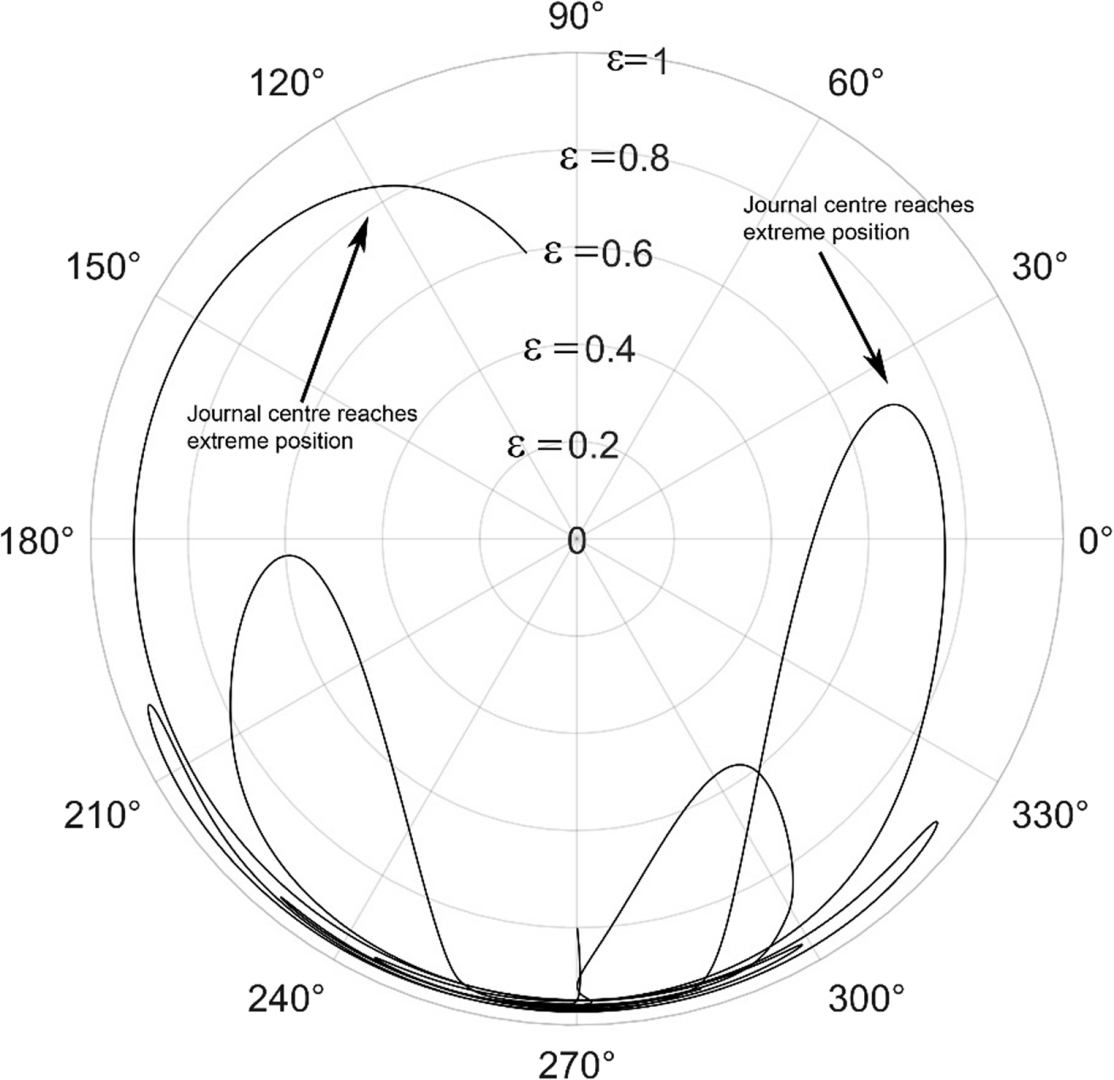
Journal centre plot for periodic load (unidirectional), Re = 4000, $\Lambda=1,\,\varepsilon=0.8,\overline M=5,\,\Omega=0.5,\;\mathrm{groove}\;\mathrm{angle}=18^\circ$.

**Figure 10:**
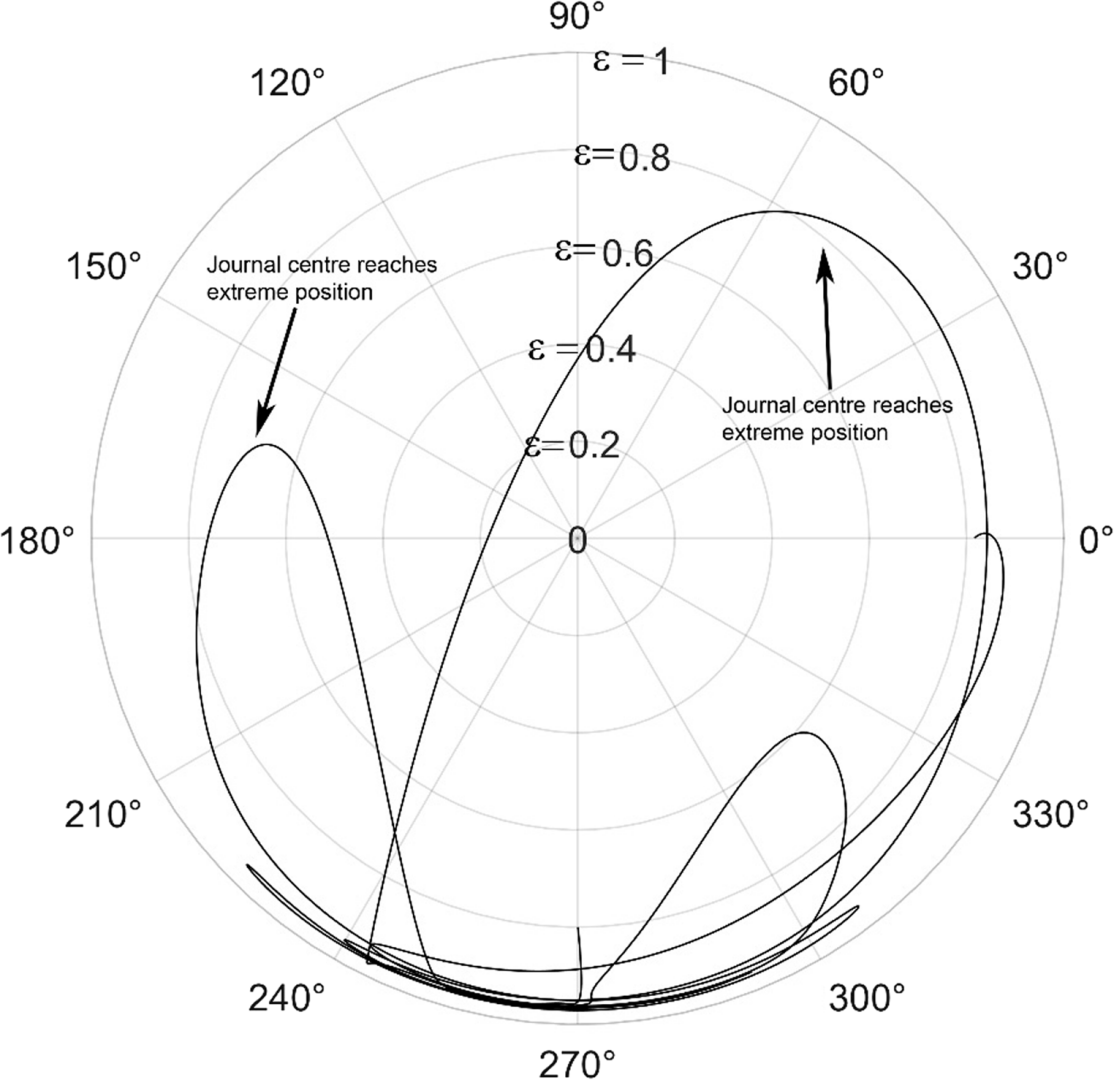
Journal centre plot for periodic load (unidirectional), Re = 16,000, $\Lambda=1,\,\varepsilon=0.8,\overline M=5,\,\Omega=0.5,\;\mathrm{groove}\;\mathrm{angle}=18^\circ$.

**Figure 11:**
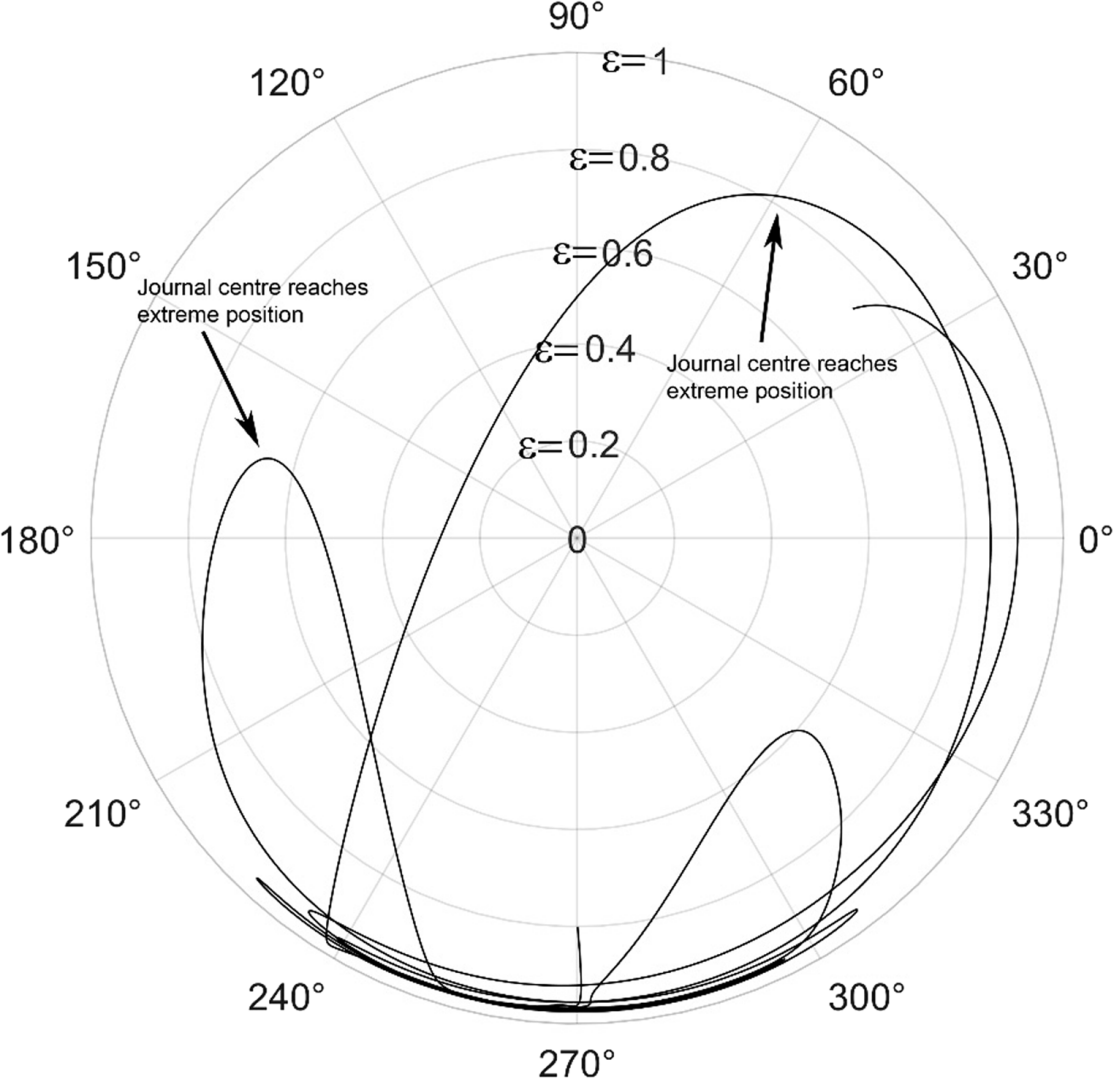
Journal centre plot for periodic load (unidirectional), Re = 55,000, $\Lambda=1,\,\varepsilon=0.8,\overline M=5,\,\Omega=0.5,\;\mathrm{groove}\;\mathrm{angle}=18^\circ$.

**Figure 12:**
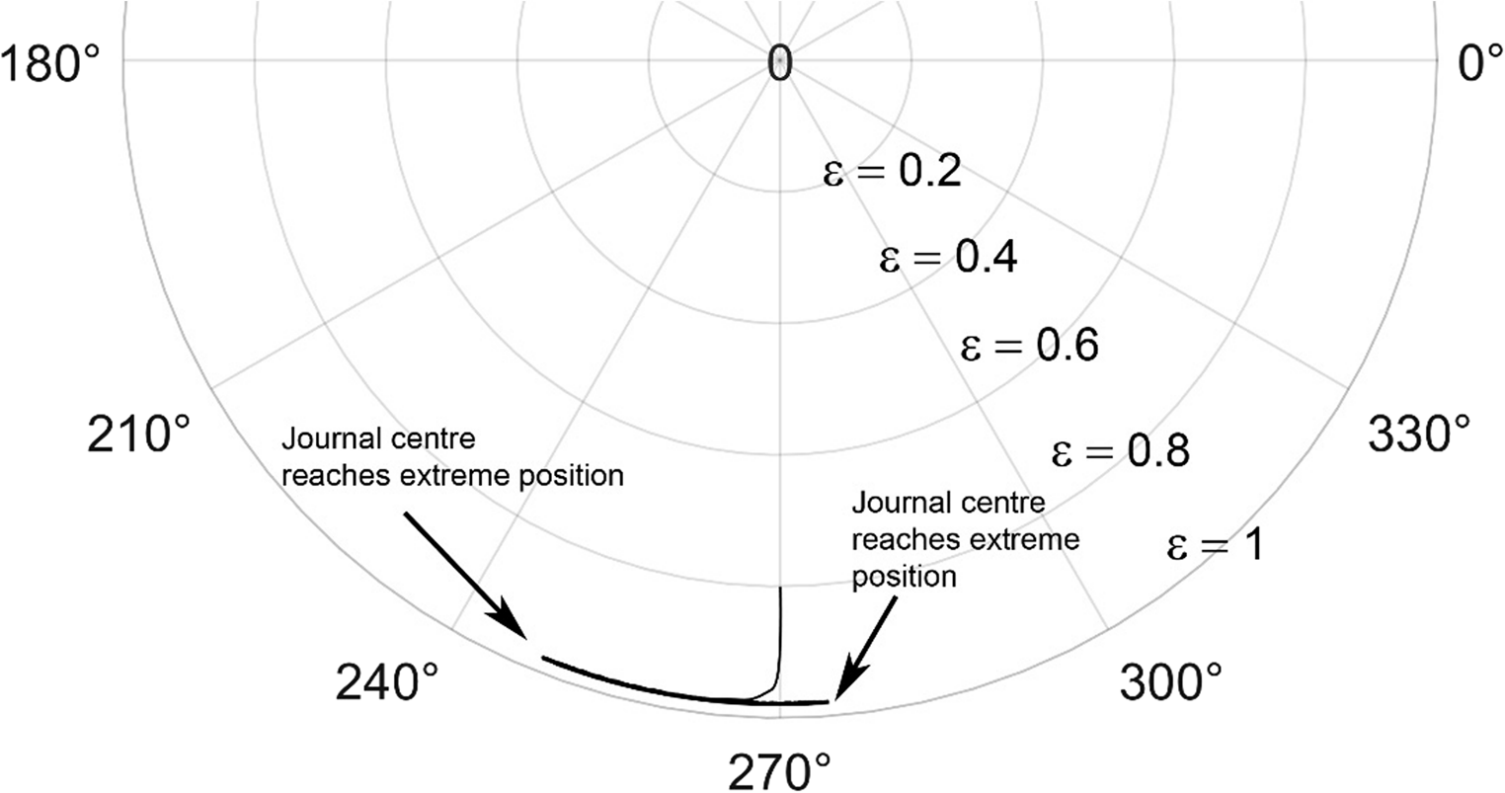
Journal centre plot for fluctuating rotating load, Re = 4000, $\Lambda=1,\,\varepsilon=0.8,\overline M=5,\,\Omega=0.5,\;\mathrm{groove}\;\mathrm{angle}=18^\circ$.

**Figure 13:**
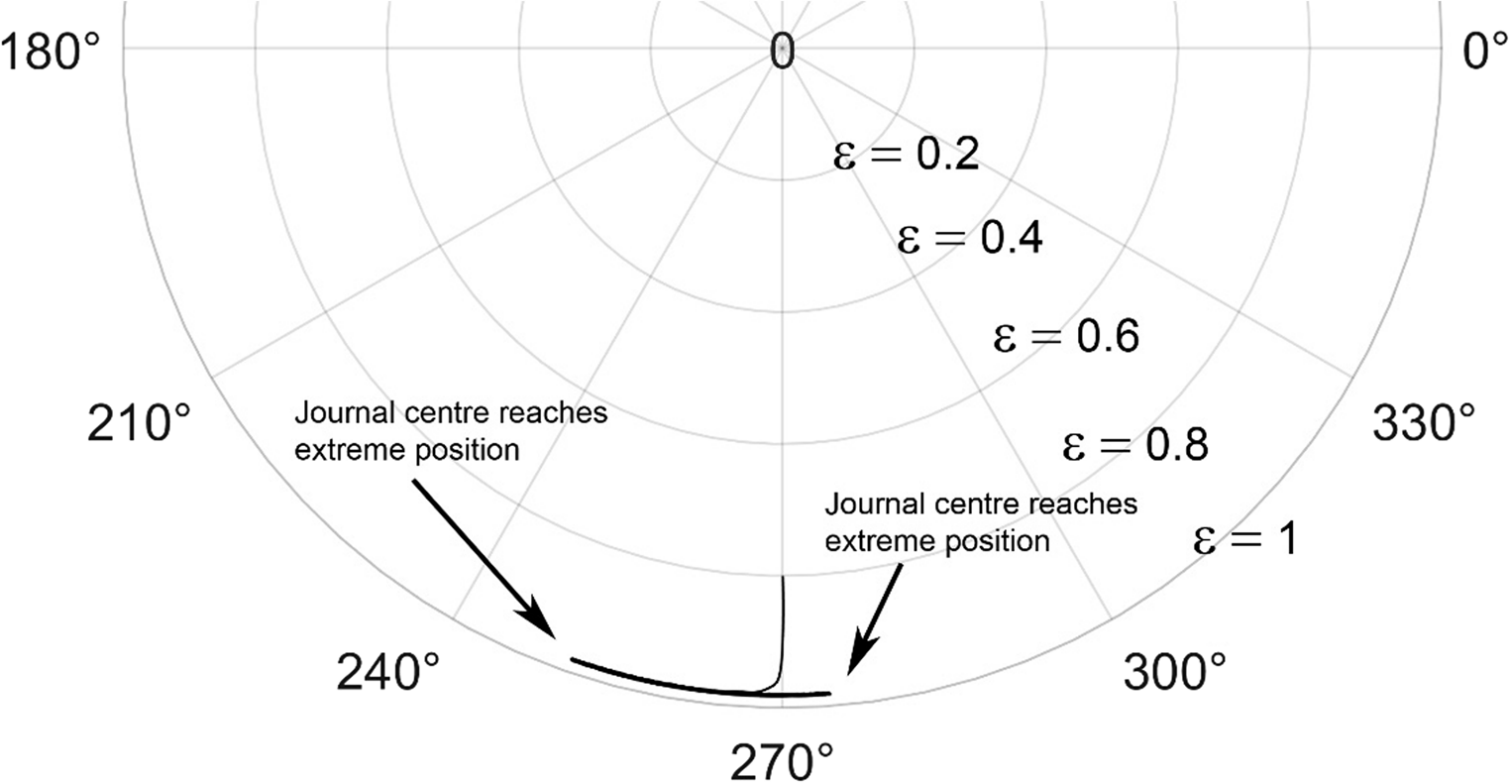
Journal centre plot for fluctuating rotating load, Re = 16,000, $\Lambda=1,\,\varepsilon=0.8,\overline M=5,\,\Omega=0.5,\;\mathrm{groove}\;\mathrm{angle}=18^\circ$.

**Figure 14:**
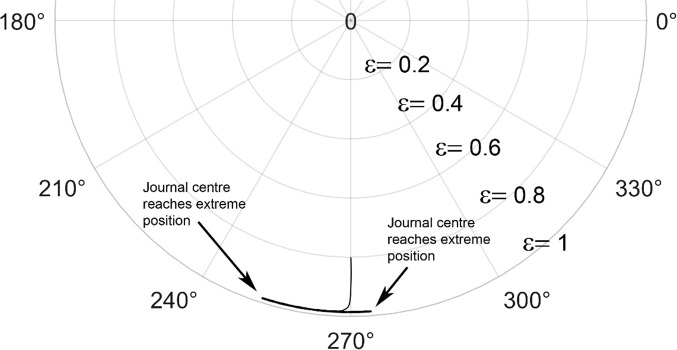
Journal centre plot for fluctuating rotating load, Re = 55,000, $\Lambda=1,\,\varepsilon=0.8,\overline M=5,\,\Omega=0.5,\;\mathrm{groove}\;\mathrm{angle}=18^\circ$.

**Figure 15:**
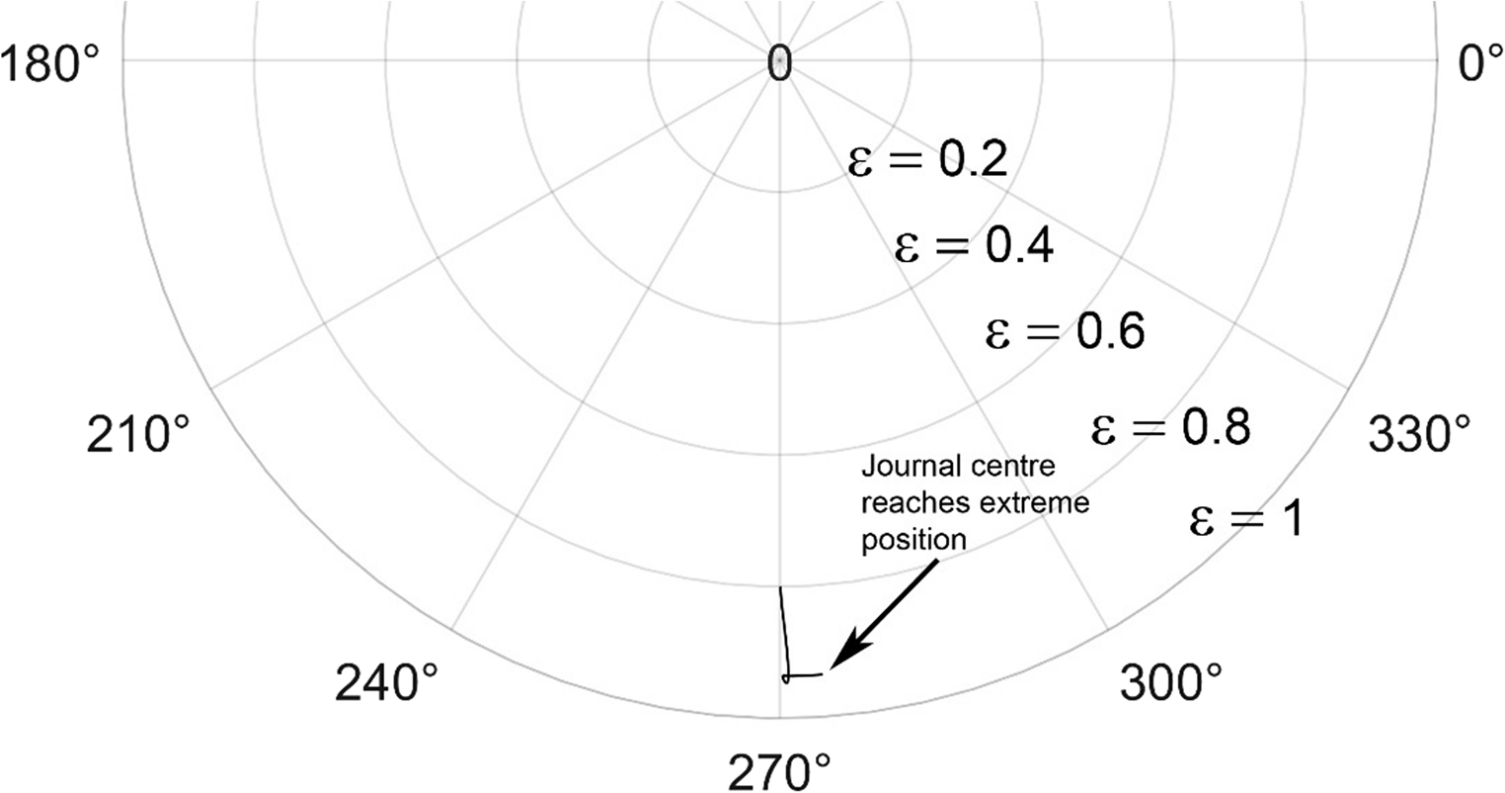
Journal centre plot for constant load (unidirectional), Re = 4000, $\Lambda=1,\,\varepsilon=0.8,\overline M=5,\,\Omega=0.5,\;\mathrm{groove}\;\mathrm{angle}=36^\circ$.

**Figure 16:**
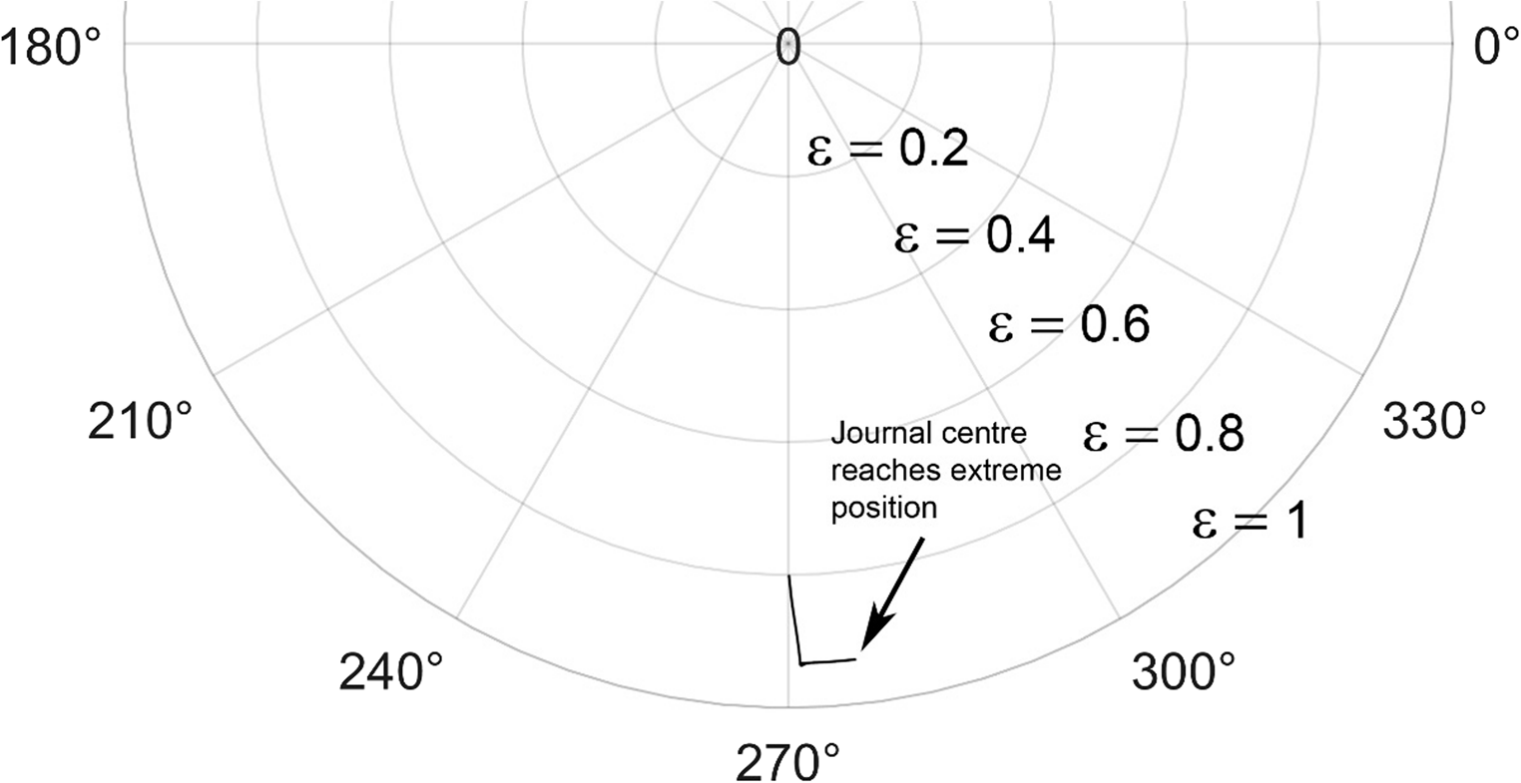
Journal centre plot for unidirectional constant load (unidirectional), Re = 16,000, $\Lambda=1,\,\varepsilon=0.8,\overline M=5,\,\Omega=0.5,\;\mathrm{groove}\;\mathrm{angle}=36^\circ$.

**Figure 17:**
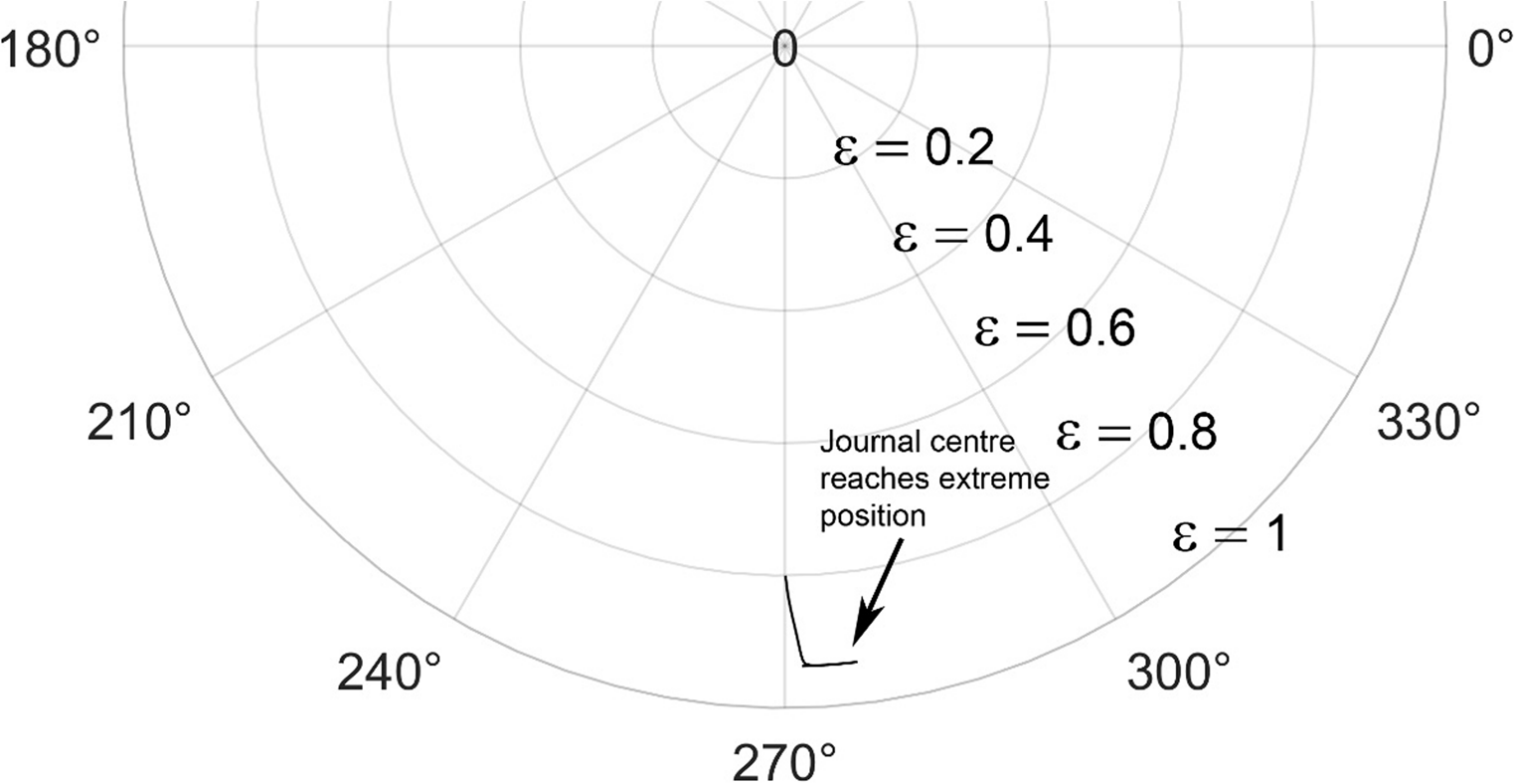
Journal centre plot for constant load (unidirectional), Re = 55,000, $\Lambda=1,\,\varepsilon=0.8,\overline M=5,\,\Omega=0.5,\;\mathrm{groove}\;\mathrm{angle}=36^\circ$.

**Figure 18:**
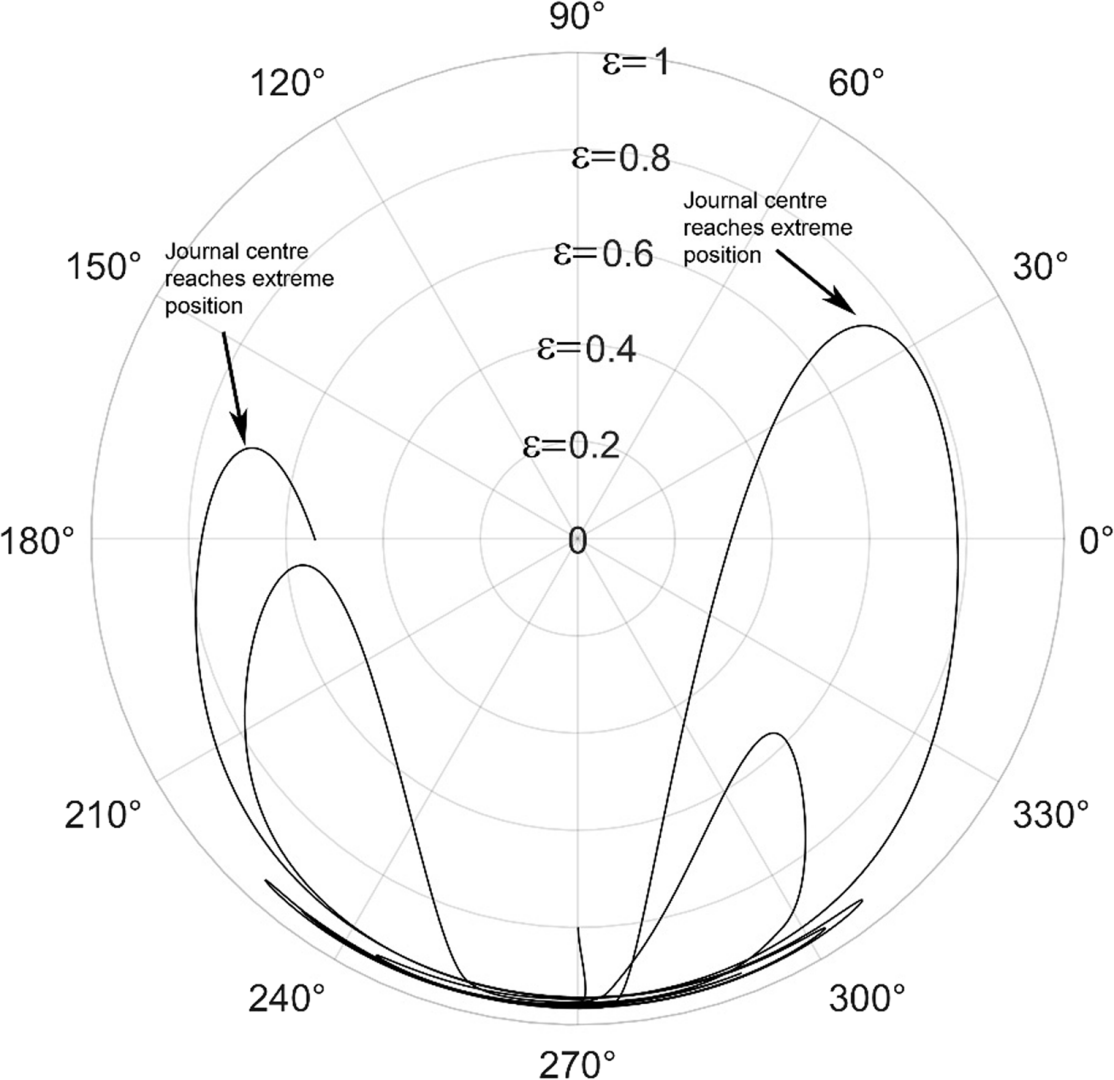
Journal centre plot for periodic load (unidirectional), Re = 4,000, $\Lambda=1,\,\varepsilon=0.8,\overline M=5,\,\Omega=0.5,\;\mathrm{groove}\;\mathrm{angle}=36^\circ$.

**Figure 19:**
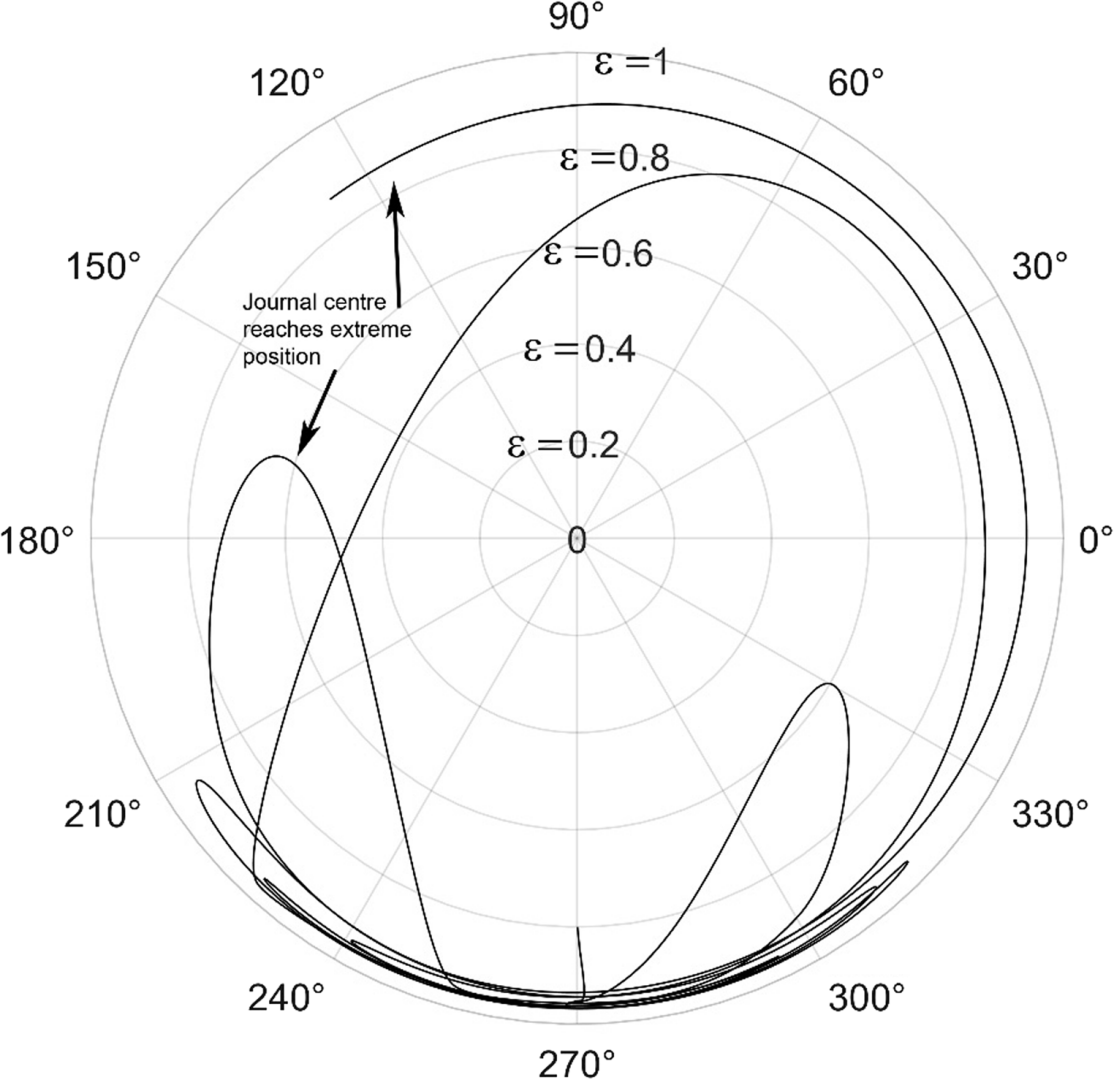
Journal centre plot for periodic load (unidirectional), Re = 16,000, $\Lambda=1,\,\varepsilon=0.8,\overline M=5,\,\Omega=0.5,\;\mathrm{groove}\;\mathrm{angle}=36^\circ$.

**Figure 20:**
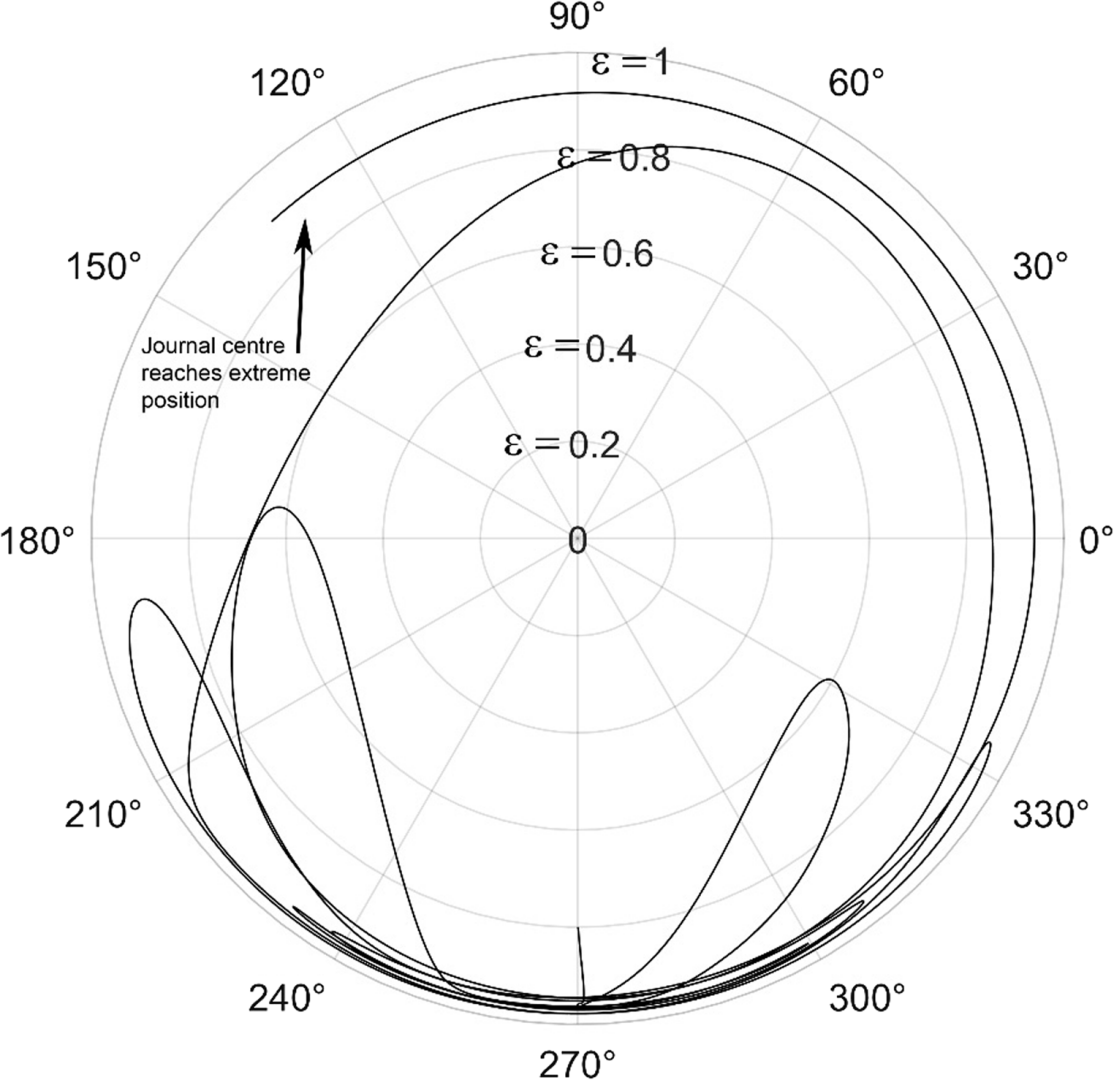
Journal centre plot for periodic load (unidirectional), Re = 55,000, $\Lambda=1,\,\varepsilon=0.8,\overline M=5,\,\Omega=0.5,\;\mathrm{groove}\;\mathrm{angle}=36^\circ$.

**Figure 21:**
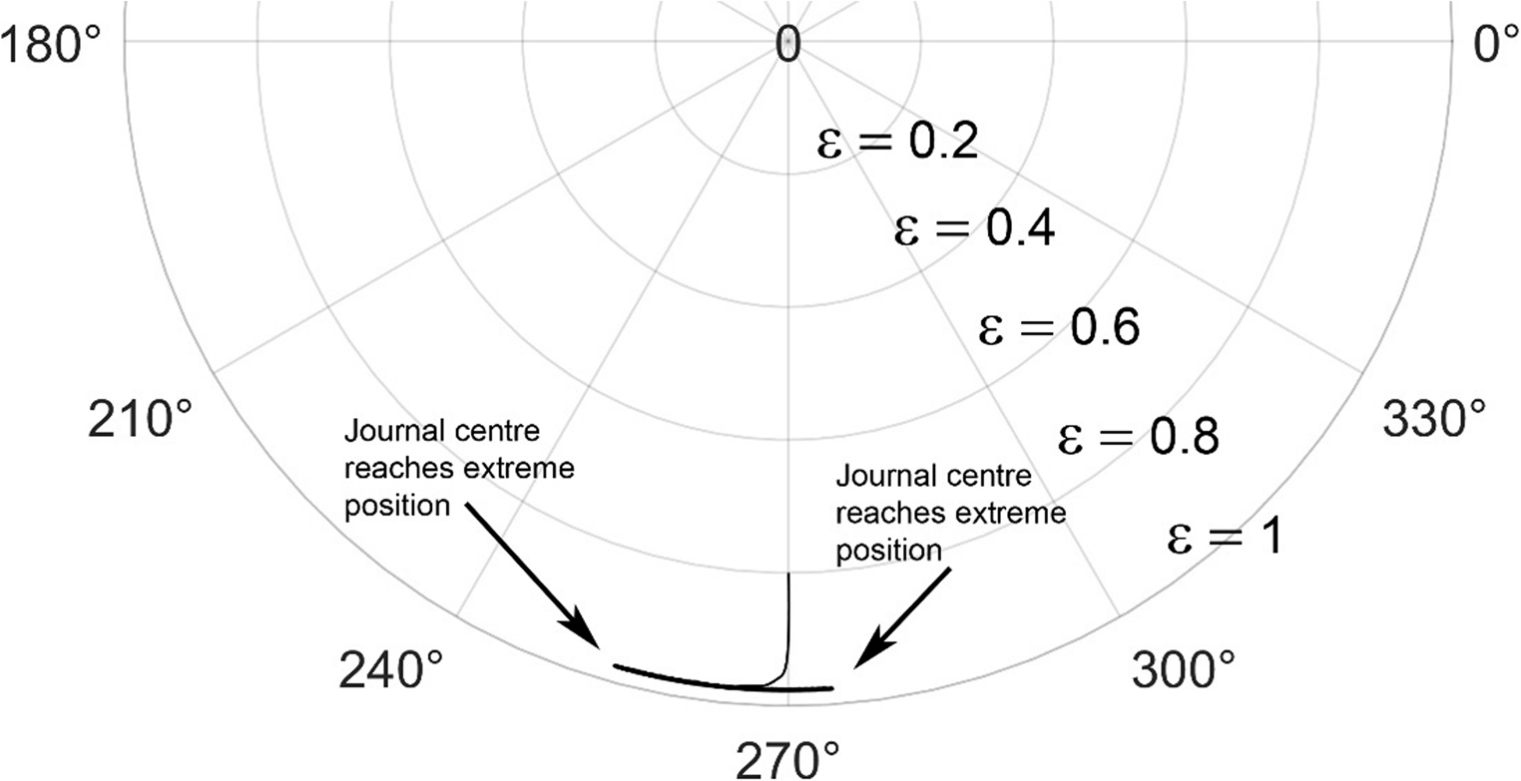
Journal centre plot for fluctuating rotating load, Re = 4,000, $\Lambda=1,\,\varepsilon=0.8,\overline M=5,\,\Omega=0.5,\;\mathrm{groove}\;\mathrm{angle}=36^\circ$.

**Figure 22:**
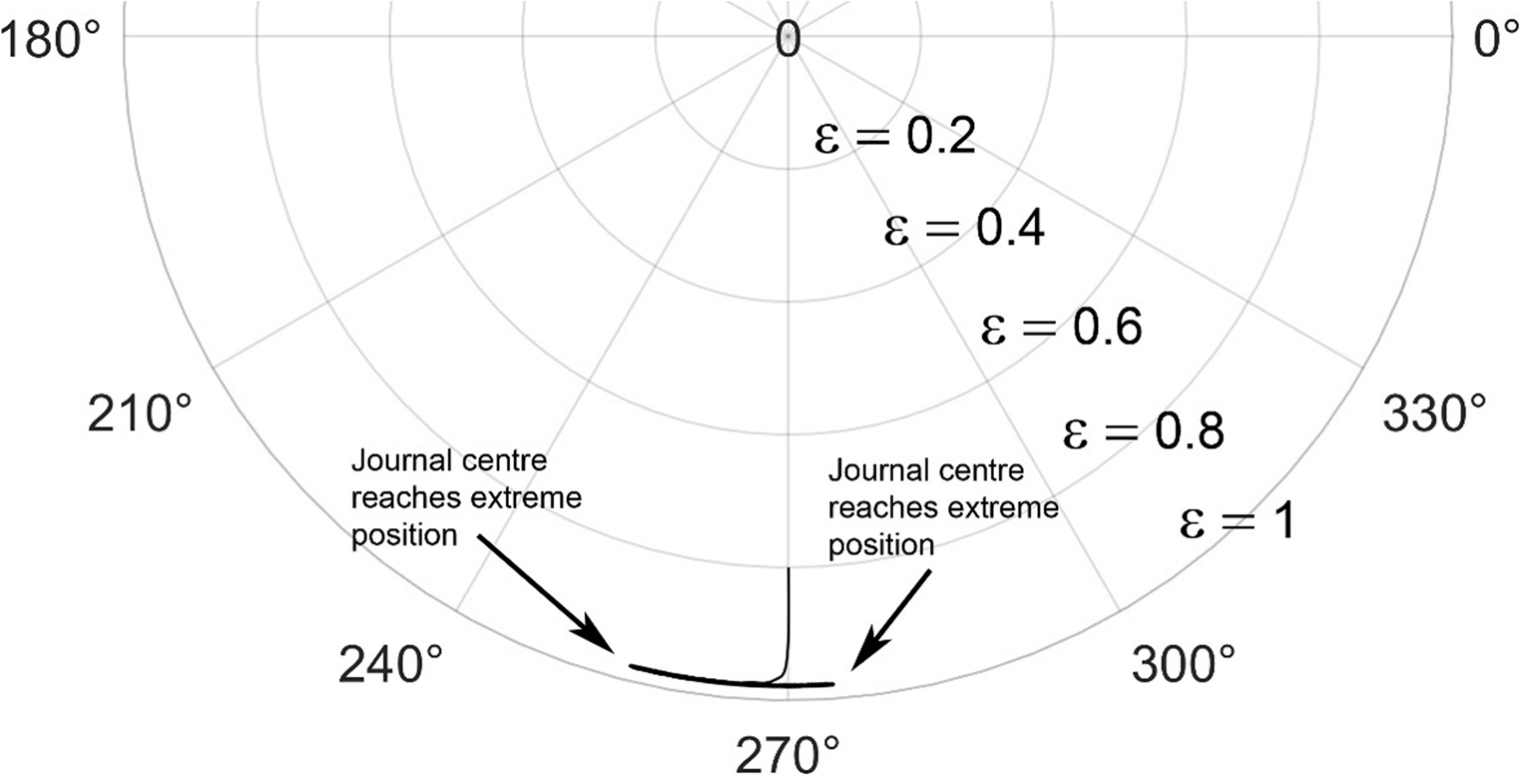
Journal centre plot for fluctuating rotating load, Re = 16,000, $\Lambda=1,\,\varepsilon=0.8,\overline M=5,\,\Omega=0.5,\;\mathrm{groove}\;\mathrm{angle}=36^\circ$.

**Figure 23:**
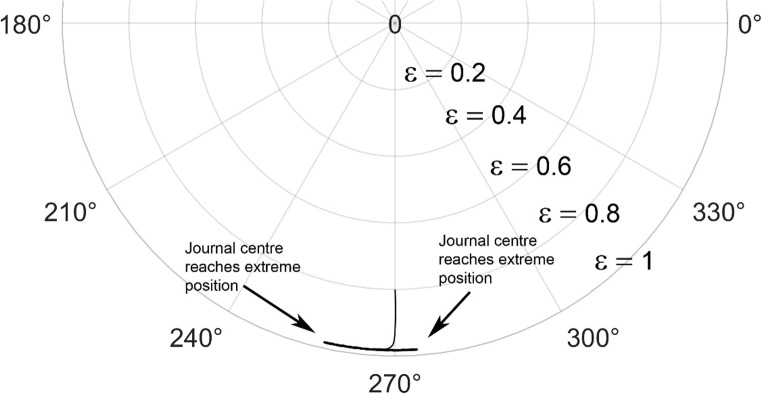
Journal centre plot for fluctuating rotating load, Re = 55,000, $\Lambda=1,\,\varepsilon=0.8,\overline M=5,\,\Omega=0.5,\;\mathrm{groove}\;\mathrm{angle}=36^\circ$.

Figures 6, 7, 8 and Figs. 13, 14 and 15 show the locus of the journal when subjected to constant loading for 18^o^ and 36^o^ groove angles respectively. The journal locus converges to a stable position for the turbulent regime considered (Re = 4000 to 55,000). It can be witnessed that the path length traced by the journal center reduces as the fluid regime changes from laminar to turbulent. The journal locus for unidirectional periodic loading is shown in the Figs. 9, 10 and 11 for groove angle 18^o^ and Figs. 18, 19 and 20 for groove angle 36^o^. The trajectory of the journal centre is longer when compared to the locus plotted using constant load for both groove angles. For 18^o^ groove angle, the path traversed is large and reduces as the value of the Reynolds number is increased. A similar pattern is seen for 36^o^ groove angle. Though the trajectory of the shaft centre does not converge to a specific point as in constant loading, the locus does not come in contact with the clearance circle indicating stability of the system. The journal locus for varying rotating load is plotted in the Figs. 12, 13, 14 and in Figs. 21, 22, 23 for groove angles 18^o^ and 36^o^ for Re = 4000 to 55,000. The journal center traverses extensively for both the groove angles. The path traced does not change as widely as for the periodic load condition and is similar to constant loading condition. The journal locus does not converge to a point as in the case of constant loading. The journal center path length reduces as the Reynolds number is increased similar to the constant loading condition. The journal center locus does not converge to a point, but it is confined to a specific region within the clearance circle. A similar development is noticed for both bearings with the groove angles 18^o^ and 36^o^. From the theoretical analysis perspective, it can be perceived that, with the increase in the Reynolds number, the dynamic stability of the bearing system has improved.

## Conclusion

The journal centre trajectory for Reynolds number 4000 to 55,000, under three types of loading is analysed. Journal bearing with axial groove, with angles 36^o^ and 18^o^ are considered. It is observed that when the flow conditions of the lubricant altered from laminar to turbulent, the stability of the bearing showed improvement. The journal centre locus plots for unidirectional constant loading show the path travelled by the journal centre reduces and reaches a stable position as the Reynolds number value is increased. When journal is loaded with periodic load, the journal centre traverses longer path before reaching the stable position in the laminar flow regime. The journal locus traverse reduced as the flow regime changed to turbulent. For rotating load with varying magnitudes, the journal centre trajectory retraces the path and the path length is reduced as the flow conditions changed from laminar to turbulent. For all the loading conditions, the journal locus plots show that, the bearing stability improved under turbulent conditions for all the three loads considered and for both the groove angles.

## Nomenclature

*e* eccentricity (m)

*C* Radial clearance (m)

*D* Bearing diameter (m)

$F_r,\;F_\theta$ Hydrodynamic forces (Steady state) (N)

${\overline F}_r=F_r/LDp_s$ Non – dimensional film force

${\overline F}_r$ Non – dimensional steady state hydrodynamic force

${\overline F}_\theta=F_\theta/LDp_s$ Non – dimensional steady state hydrodynamic force

*h* Fluid film thickness (m)

$\overline h=h/C$ Non – dimensional fluid film thickness

$$k_\theta,\;k_x,\;k_z,\;k_{zz}$$ Turbulence coefficients

*K1, K2, K3, K4* Slope parameters in RK method

*L* Bearing length (m)

*M* Rotor mass (kg)

$$\overline M=MC\omega^2/LDp_s$$ Mass parameter

$$n_x,n_z$$ Turbulence coefficients

*p* Fluid film pressure (Pa),

$\overline p=p/p_s$ Non – dimensional fluid film pressure

*ps* Lubricant supply pressure (Pa)

*R* Radius of journal/shaft (m)

*R*_e_ Reynolds number (Global)

*R*_*eL*_ Reynolds number (Local)

*t* time (s)

*U* Journal/Shaft velocity (m/s)

${\overline W}_\circ=\frac{W_\circ}{LDp_s}$ Non-dimensional steady state load

$\overline z=z/L$ Non – dimensional coordinates

## Greek

$$\varepsilon$$ Eccentricity ratio

$\varepsilon^\backslash$ Mid plane eccentricity ratio

$$\eta$$ Viscosity (Pa-s)

$\theta$ Non – dimensional coordinates, *θ = x / R*

θ^*^ Coordinate in the circumferential direction

$$\lambda ,\Omega$$ Whirl ratio $$\lambda = {\omega_p}/\omega ,\,\,\,\Omega = {\omega_p}/\omega \,$$

$$\Lambda$$ Bearing number $$\Lambda = 6\eta \omega \,\,\,\,/\,\,{p_s}{\left( {C/R} \right)^2}$$

$\tau$ time (Non – dimensional)

$\phi$ Attitude angle of the bearing (rad)

$\psi$ Assumed attitude angle of the bearing (rad)

$\omega$ Journal/shaft rotational speed (rad/s)

${\omega_p}$ Frequency of journal/shaft vibration (rad/s)

## Data Availability

MATLAB source code and the data that support the findings of this study are available from the first [R.M.] and the corresponding author, [S.S.B.], upon reasonable request.
